# Toughening Mechanisms in Stacked Bilayer Graphene Sheets by Means of Sandwiched 1D Nano-rebars

**DOI:** 10.1021/acs.nanolett.5c01513

**Published:** 2025-04-21

**Authors:** Muhammad Usama Arshad, Yuxiang Gan, Congjie Wei, Xingkang She, Pavan V. Kolluru, Chenglin Wu, Mohammad Naraghi

**Affiliations:** % Department of Materials Science and Engineering, Texas A&M University, College Station, Texas 77843, United States; † Department of Civil and Environmental Engineering, Texas A&M University, College Station, Texas 77843, United States; & Department of Aerospace Engineering, Texas A&M University, College Station, Texas 77843, United States

**Keywords:** graphene, 1D−2D nanomaterials, conformity, crack growth resistance

## Abstract

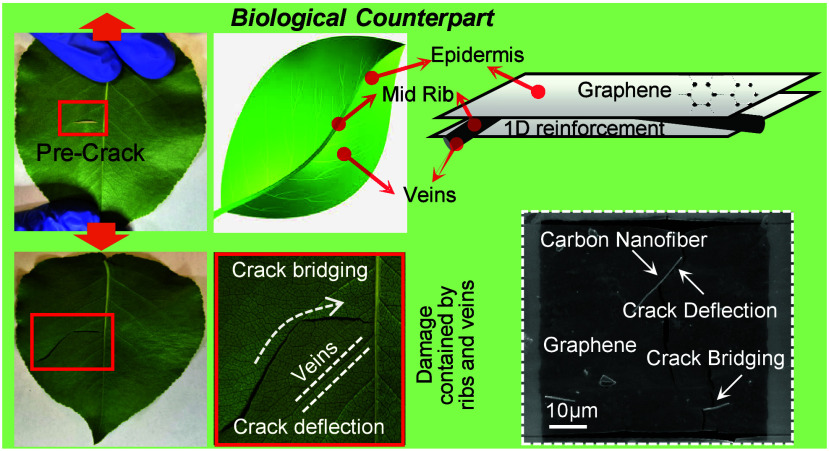

Graphene, with an intrinsic strength of >100 GPa, exhibits promise for armor applications. However, the thermodynamically favorable manufacturing defects severely diminish their achievable strength. To remedy this, 1D nano-rebars, sandwiched between graphene monolayers, were studied. Real-time mode-I crack growth resistance was studied in reinforced graphene under SEM, which revealed that the dissipative interactions between 1D and 2D nanomaterials can increase the ductility of graphene by over 100%, albeit at a slight loss in effective toughness. This significant improvement was analyzed by introducing the concept of geometric conformity to explain the load transfer between them. By means of finite element analysis and shear lag models we explained the contribution of dissipative interactions between nano-rebar and graphene in reducing stress concentration around cracks, leading to high ductility. The dissipative bonds were found to be more favorable over covalent bonds in terms of maintaining a lower interface stress, further delaying interface local failure.

Lightweight, strong, and tough materials are in high demand to protect valuable assets, such as electronic devices, vehicles, and people against external loads.^1−4^ Two-dimensional (2D) nanomaterials are favorable candidates owing to their exceptional physical properties.^5−7^ Among myriads of nanomaterials, one-atom-thick sheets of carbon atoms, graphene, with an intrinsic strength as high as 100–130 GPa, stand out.^8−10^

Despite the demonstrated outstanding properties of graphene, its mechanical strength is significantly compromised by defects such as pinhole defects, Stone–Wales defects, grain boundaries, and edge defects.^11^ For instance, 1–2 atom vacancies may lower the strength by 14–17.7%, and Stone–Wales defects reduce their fracture strength by >50% in the zigzag direction.^12−14^ Moreover, manufacturing defects are thermodynamically favorable due to several reasons, e.g., increase in entropy, relaxation of strain, and energetic stability.^15^ Thus, production of large-scale graphene while maintaining its strength is still challenging.^16−18^ This challenge may be addressed through improved manufacturing or by reinforcing the graphene with other materials.^19−21^ Given the thermodynamics laws that promote the formation of defects, the latter seems to be a more promising route.

Cao et al.^22^ spin coated silver nanowires (AgNWs) on graphene to enhance the mechanical and electrical properties. A recent work by Li et al.^23^ identified the length of the nanowires as the key to switch between failure modes of nanowire pull out and failure. Hacopian et al.^24^ integrated carbon nanotubes (CNTs) with graphene by growing them together via chemical vapor deposition (CVD). The integration leveraged a synergistic effect where CNTs reinforced the graphene, increasing its load-bearing capacity and energy absorption. Despite that, the cofabrication of 1D and 2D materials restricts the type of one-dimensional (1D) rebars.

Improving the fracture toughness of graphene by adding reinforcing nano-rebars is highly dependent upon the ability of the interface between them to transfer loads. The shear load transfer is controlled by the interplay between 1D–2D surface energy and internal strain energies in 2D nanomaterials in the vicinity of reinforcements.^25,26^ Improving load-transfer via controlling the surface energies of the 1D–2D interface (e.g., covalent bonding between rebar and graphene) may come at the cost of disrupting the sp^2^ hybridization of bonds in graphene, thereby lowering its strength.^27^ Other approaches include surface functionalization of the rebar to promote secondary bonds.^28^ As a consequence of improved chemical or physical bonds between graphene and 1D rebars, graphene with its low out-of-plane bending stiffness may conform more effectively around the 1D rebars. This geometric conformity, i.e., increased contact area between them, promotes the shear load transfer between them, while nonconformal configurations can result in weak interfaces, thereby compromising the mechanical integrity of the composite and potentially leading to premature failure. In an extreme case, when graphene is fully wrapped around rebars, in-plane stresses may grow in graphene, which may offset the gains in toughness achieved via 1D rebar.

In this letter, we demonstrate that the embedded nano-rebars in-between the stacked bilayer graphene (SBLG)^29^ which interacts with the graphene via van der Waals (vdW) forces drastically improve the resistance of graphene to crack growth, Figure 1(a–f). The fabrication of SBLG as explained in our earlier work^29^ allows for the sandwiching of other nanomaterials as strengthening rebars. The sample studied here is composed of two layers of CVD-grown graphene, which sandwich individual carbon nanofibers (CNFs) made via pyrolysis of polymeric nanofibers. Each of the layers and CNFs are manufactured separately and then stacked together. Therefore, the manufacturing method is highly versatile. The complete step-by-step fabrication route and explanation are presented in Figure S1. Figure 1(f) displays the post-mortem image of a CNFs/SBLG sample, where CNFs are located ahead of the cracks. The crack suppression and deflection implemented in SBLG via embedded nano-rebars are similar to those at play in tree leaves, in which the leaf epidermis and veins/midribs are respectively analogous to graphene and nano-rebars, Figure 1(g1–g3).

**Figure 1:**
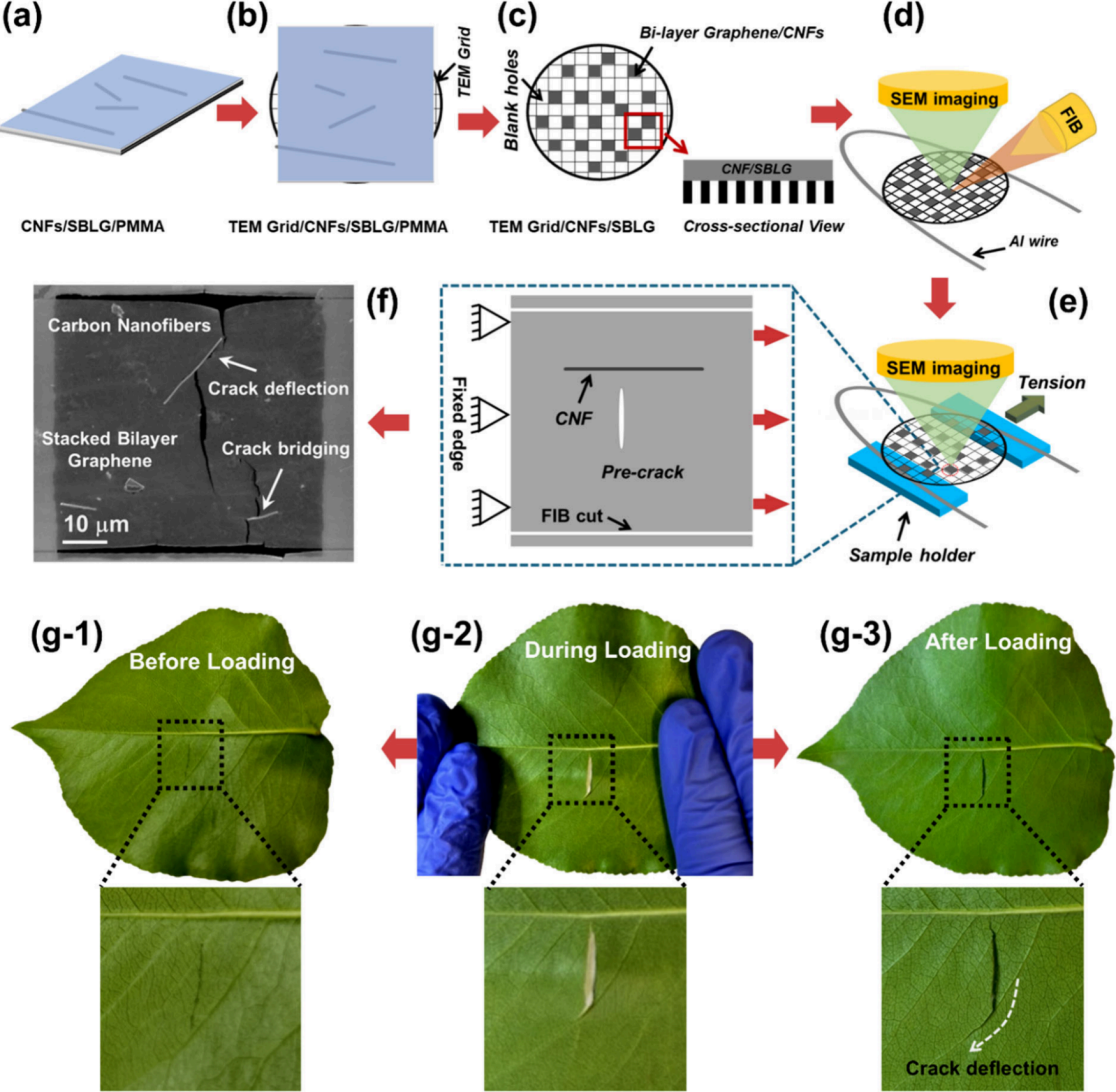
Schematic diagram of the fabrication and mechanical characterization of SBLG/CNFs: (a–e) step by step SBLG/CNFs sample manipulation for mechanical testing, (f) in situ SEM observation of crack growth subjected to displacement loading of the SBLG/CNFs showing the crack deflecting and crack bridging mechanism, (g1–g3) inspiration from the tree leaf and the mechanism of crack deflection and crack bridging due to veins.

The mechanical behavior of 1D–2D structures and the contribution of 1D nano-rebars in delaying crack growth in 2D material are highly dependent on load transfer along their interfaces. The degree to which graphene conforms around the 1D nano-rebar is essential to control the load transfer between the two. For clarity, the concept of geometrical conformity is explained in Section S2 for two sheets with zero bending stiffness which sandwich a rigid cylindrical rod. As shown in the schematic in Figure 2(a), at the highest point of CNF (with respect to the flat sections of the sheets), the sheets (i.e., graphene) are in full contact with the rod (i.e., CNF), and the contact extends up to the separation point shown with a red dot. The angular position of the separation point, θ, is a measure of conformity. In theory, this value ranges from ∼0° to 90°, with 90° representing a fully wrapped graphene. The angle controls the load transfer between the CNF and graphene, as discussed in Section S6. In practice, interatomic spacing between graphene sheets is nonzero (∼0.34 nm), and we can define the point of separation between CNF and graphene to be the location at which the distance between them reaches a critical value, simply twice the interatomic spacing between graphene sheets (∼0.68 nm).

**Figure 2:**
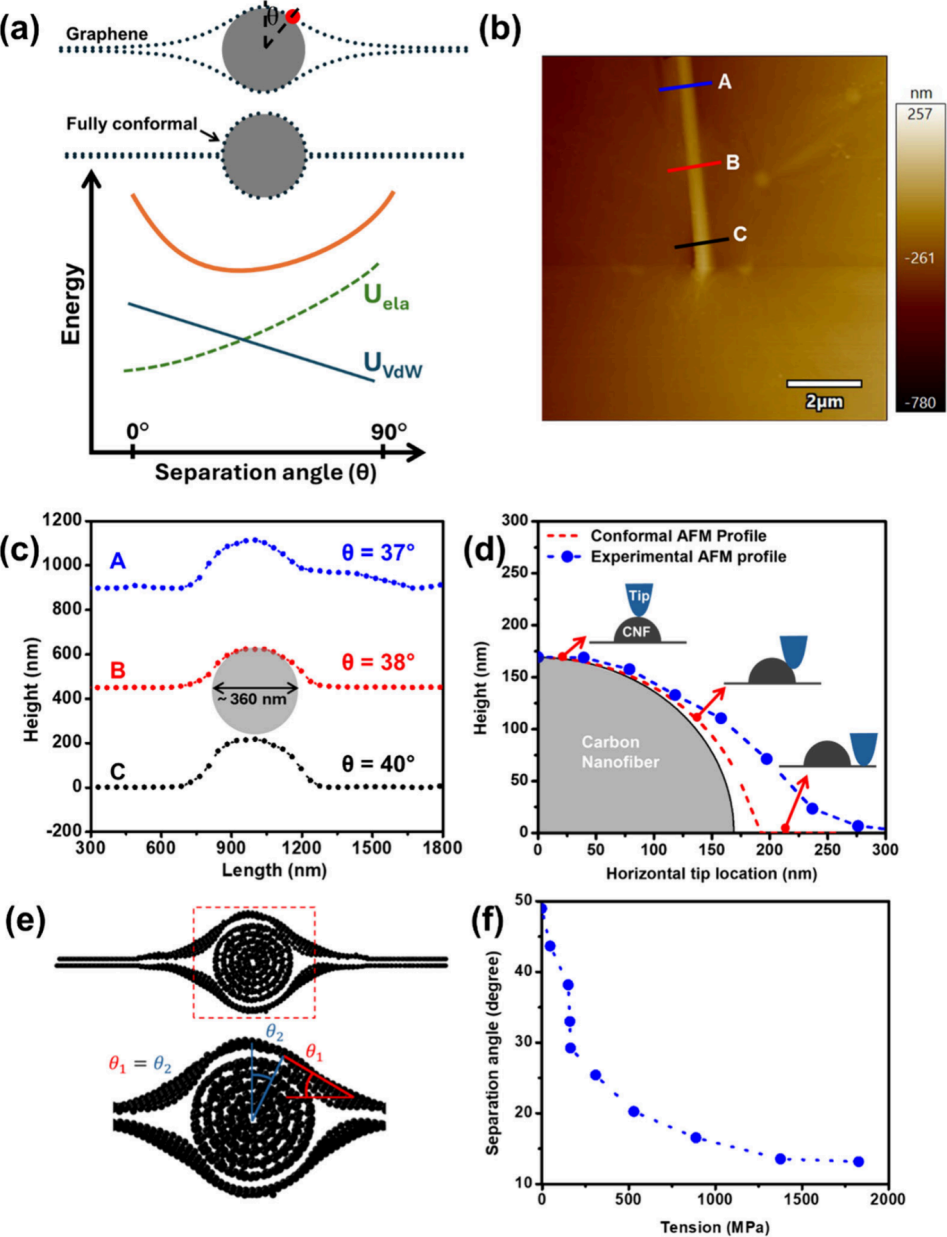
Material characterizations to estimate the level of conformity. (a) schematic diagram of fully conformal and less conformal graphene around CNF with an explanation of relationship between different energies and separation angle. (b, c) Representative AFM topography image of a CNF/SBLG composite film showing the corresponding height profiles at three distinct locations A–C along the length of the CNF. (d) Quantitative AFM line scan analysis used to show that the experimentally measured profile of graphene (dotted line) is much wider than the analytically expected height profile (solid line) for a fully conformal graphene layer even after accounting for tip geometry convolution artifacts (see SI for detailed analysis), thus confirming that graphene in CNF/SLBG composite films is nonconformal. (e) Molecular dynamic (MD) simulations of a CNF sandwiched between two graphene sheets used to validate the AFM-based characterization and evaluate (f) the separation angle vs tension in graphene for different cases (see SI for details).

Surface topography imaging via atomic force microscopy (AFM) and line scan analysis of CNF/SBLG composite films were employed to study the conformity of graphene around CNFs, Figure 2(b–d). The AFM line profile is composed of a circular arc in the center (the CNF) transitioning into a flat line away from the CNF where the two layers merge with unmediated overlap. Assuming that the CNF is symmetrically sandwiched between the two graphene layers, the vertical distance of the highest point of the AFM height profile from the flat line, Figure 2(c), is the radius of the CNF. This is in great agreement with the TEM images in Figure S3.

The line scan analysis in Figure 2(d) shows the experimentally measured AFM height profile (blue dotted line). On the same graph, we plotted the hypothetical AFM profile for a fully conformal graphene–CNF interface (red dotted line) by accounting for tip geometry convolution effects such as finite radius and conospherical tip geometry (see Section S2). As shown in Figure 2(d), the AFM profile is considerably wider than the hypothetical plot for conformal overlaps, demonstrating that the graphene is not fully conformal around the CNF (i.e., θ < 90°).

A least-squares regression analysis of the experimentally measured AFM height profiles of graphene from the as-prepared CNF/SLGB composites (Figure 2(b–d)) shows that the separation angle θ between graphene and CNF in the undeformed (i.e., ∼zero applied tension) state was ∼37–40°, SI Section S2.1, in agreement with MD simulations, Figure 2(e,f).

The conformity of graphene around 1D reinforcement materials in SBLG-CNF represents a balance between surface energy and internal strain energy in graphene. On one hand, graphene may fully envelop 1D reinforcements (each sheet covering half of the CNF circumference) to minimize surface energy. On the other hand, internal elastic energy in graphene penalizes in-plane stretching required for the out-of-plane wrapping, i.e., favoring lower separation angles. This is especially true when graphene is held in place at its boundaries during manufacturing (in our experiment). While it is not trivial to assess the magnitude of stresses in graphene experimentally, in a handful of samples we observed failure of one of the sheets in the vicinity of CNFs, which points to a nonzero in-plane stress, Figure S3(a). In most cases, the stresses are not large enough to cause failure; see Figure S3(b,c). The balance of these two mechanisms is schematically shown in Figure 2(a).

Further insight into the interplay between elastic energy and conformity was obtained via MD simulations using LAMMPS;^30^ see Figure 2(e) and Section S2.3. Figure 2(f) presents the relationship between the separation angle and tension in graphene estimated from the MD simulation. For the separation angles of ∼37–40° from AFM images, MD simulations point to a tension of 152.40–115.35 MPa in SBLG. As intrinsic strain energies increase, the nonconformity of graphene also increases, reducing the separation angle. These nonconformal arrangements create gaps, which diminishes load transfer. Further insight into the morphology of graphene and CNF was obtained via Raman spectroscopy, as explained in Section S2.4, pointing to a low defect density in graphene.

We then studied crack growth in composite graphene by subjecting precracked samples to far-field elongation (mode I fracture). The experimental procedure is described in an earlier work.^29^ In these experiments, we did not record the force directly applied on graphene, although the stresses were estimated based on the measured strain and known elastic moduli of each.^31,32^

The resistance of graphene to crack growth with and without CNFs was studied by measuring the critical elongation at the onset of crack growth (δ_*C*_). Figure 3(a,b) are, respectively, the low- and high-magnification SEM images during the fracture test in one of the 7 samples that were tested and reported. Before applying any load, low- and high-magnification SEM images were captured as the reference image. For the sample shown in Figure 3(a,b), graphene was 68.75 μm long and 33.70 μm wide, the CNF length (*L*_*CNF*_) was 62.30 μm, and a 3.60 μm long FIBed precrack was cut into the sample near the CNF.

**Figure 3:**
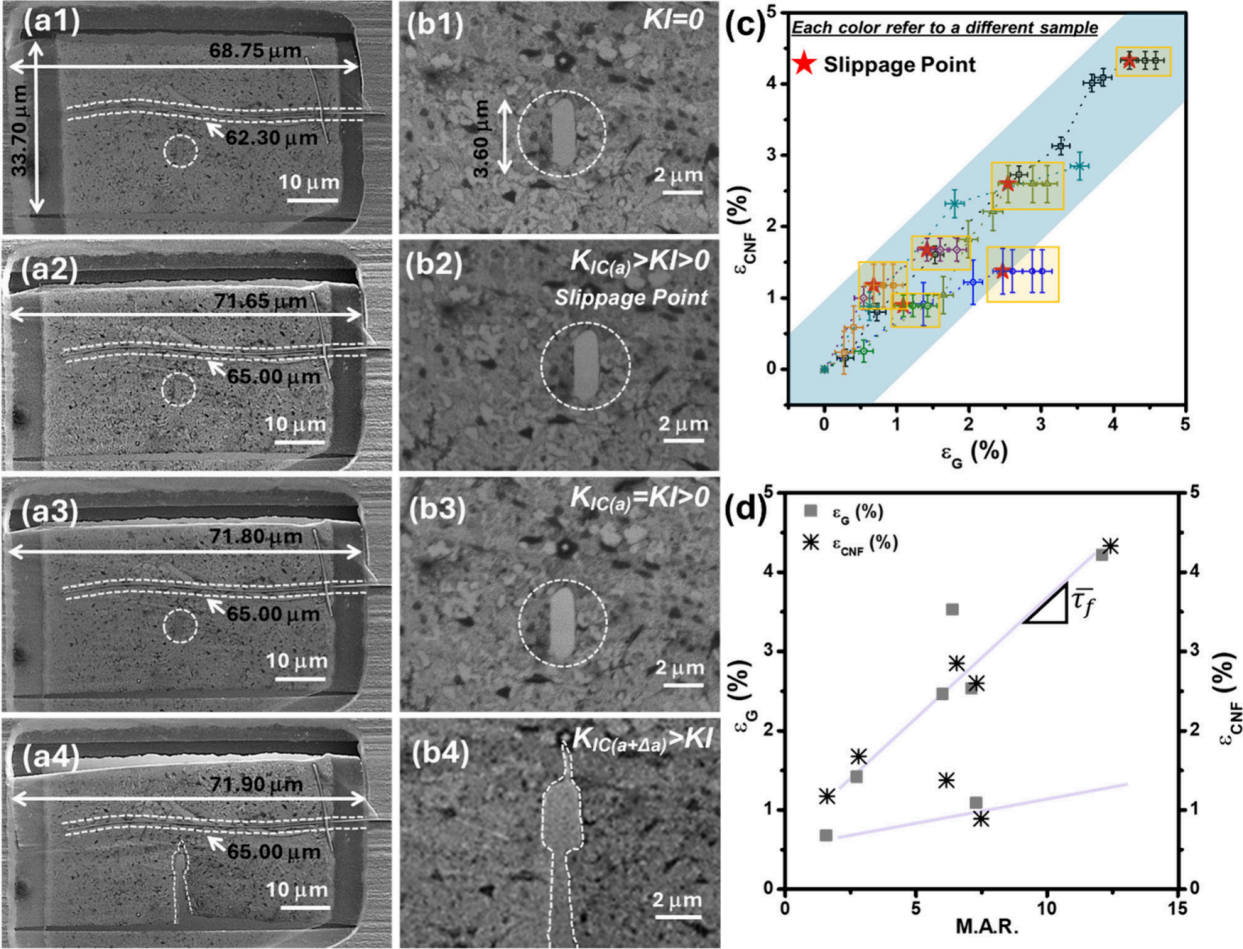
In situ SEM images of tensile testing at different steps and magnifications, (a1–a4) before applying a load, at the onset of slippage, at the onset of crack propagation, and after crack propagation, respectively, (b1–b4) high-magnification SEM images of the corresponding tensile test steps, (c) relationship between strain of the CNF and graphene, (d) relationship between the strain of the CNF and graphene vs modified aspect ratio of the CNF.

These images were used to calculate the elongation of each CNF/SBLG. The effective strain on graphene, calculated from the grip displacement, was equal to the strain measured between adjacent particles near the grips, confirming no slippage. However, in all samples, we observed that at early stages of the experiment the average strain in CNF and graphene remains the same and increases with the applied displacement, indicating affine deformation with negligible CNF sliding. This trend continues until a critical value of elongation (average strain) in graphene is reached, after which the strain in the CNF does not increase any further, while the graphene can further deform with no crack growth (the CNF starts to slide on the graphene), shown with a red star in Figure 3(c). This step continues for one or two more increments of deformation, after which the crack grows. For the example shown in Figure 3(a,b), the elongation of graphene at the point of CNF slippage was ∼2.7 μm (effective strain of ∼3.9%), while the graphene reached a strain of ∼4.43% before the crack growth. The details for the applied load and other samples are given in Section S3 and Figures S6–S11.

MD simulations using ReaxFF potential were performed to analyze load transfer between graphene and CNFs^33^ with two interaction scenarios: strong bonding between graphene and CNFs and weak vdW forces. In the strongly bonded graphene, cracks and large-amplitude wrinkles developed extending to the composite boundaries upon loading. However, vdW interactions resulted in insignificant wrinkles, Figure 4(a). Thus, vdW interfaces disperse stress more uniformly and minimize distortion, while bonded interfaces experience high stress, promoting local failure, Figure 4(b). See SI Section S4 for a more detailed analysis.

**Figure 4:**
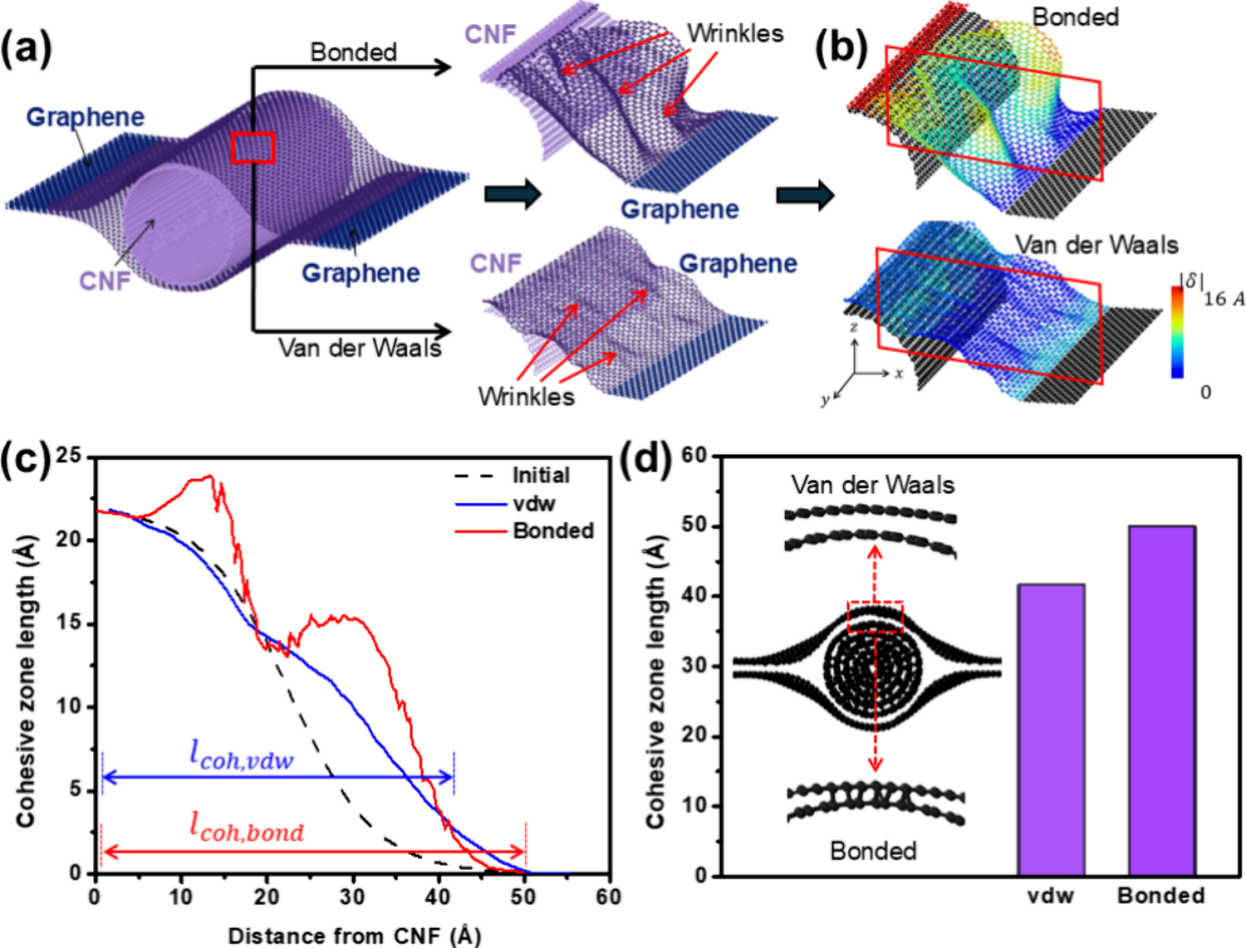
Molecular dynamic simulations. (a) Graphene–CNF–graphene structure construction and modeling results. (b) Strain contours. (c) Cross-sectional profile extracted for representative planes in part b. (d) Cohesive zone lengths.

We used finite element analysis (FEA) with the commercial software ABAQUS to investigate the impact of CNFs on the stress distribution near the crack. The properties used are provided in Section S5. The phase field analysis revealed the damage zone around the CNF, demonstrating that the CNFs shouldered a significant portion of the stress, resulting in greatly reduced stress concentration at the crack tip adjacent to the CNFs compared to the other side. Consequently, this stress distribution caused the crack to propagate from the crack tip that was away from the CNFs. Hence, the CNFs were effective in facilitating crack bridging and improving the load-bearing capacity of SBLG Figure 5(a).

**Figure 5:**
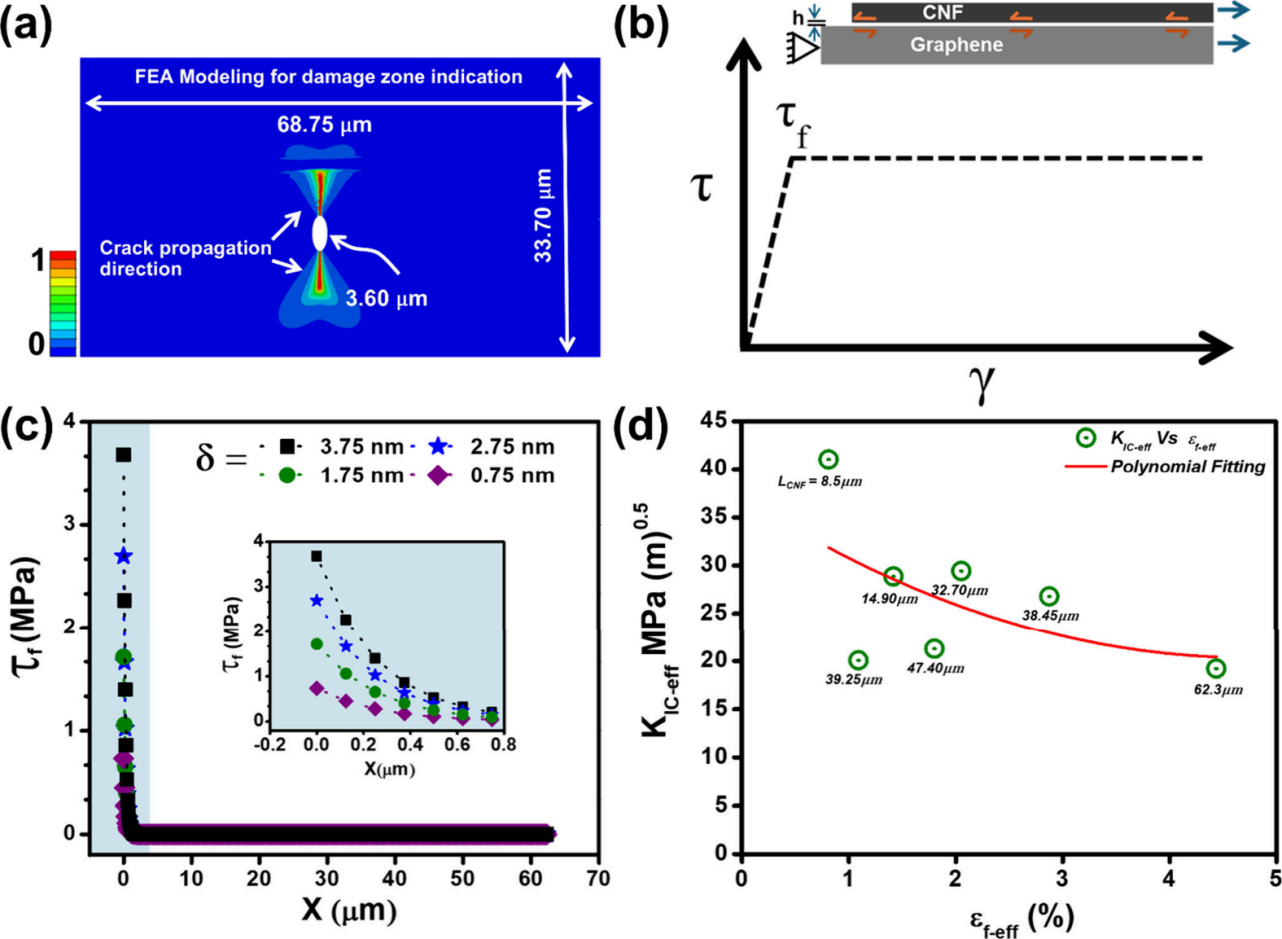
(a) Finite element phase field modeling indicating the extent of damage (0 for no damage and 1 for a fully developed crack), (b) free-body diagram of the CNF/SBLG matrix material and ideal elastic–plastic shear model for the CNF/SBLG composite, (c) stress distribution along the overlapped length for estimation of true sliding displacement (δ_*S*_) and other displacements lower than the inset image of the zoomed version of stress distribution in the highlighted area, (d) influence of CNF length on the fracture toughness and its relationship as a function of effective strain at failure of the composite.

One of the major findings of the FEA is the realization that the stress concentration caused by the cracks fades around the CNFs. We used a shear lag model to study the stress field in the CNF graphene and their interface.^34,35^Figure 5(b) shows the free-body diagram of the CNF/SBLG composite structure. In this model, “*h”* represents the composite thickness (see Section S6). A tensile normal stress, σ_0_, is applied at the right end of both the CNF and SBLG. The left end of the SBLG is fixed. The boundary conditions and sample dimensions are presented in the SI (Figure S14). The shear stress between the CNF and graphene is assumed to follow elastic–perfectly plastic behavior. In the shear lag model (see SI Section S6 for complete theoretical analysis), the normal stress, σ_0_, increases from zero. As shown in Figure 5(c), shear stress is initially concentrated at the free end of the CNF, while the majority of the interface is nearly traction free. As σ_0_ reaches a critical value, the interface along the CNF free end starts to yield. By further increasing σ_0_, the yielded interface propagates from the free end of the CNF toward the other end until the whole interface has yielded. After that, the interface between the CNF and graphene is not stable and the CNF slides. Through the shear lag model, we related the critical average strain of the CNF (the same as the strain in graphene) to the overlap length between the CNF and graphene. The strain is (Section S6.2)
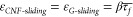
1

This equation relates the critical sliding strain to the interface shear strength. Here,  is the nondimensionalized shear strength defined as  and modified aspect ratio (M.A.R.) , measured from the experiments. To the best of our knowledge, this is the first time this type of experiment has been used to assess the shear strength of two nanomaterials. The only variables among different samples are the CNF length and SBLG width. Therefore, as shown in Figure 3(d), the *ε*_*CNF*-*sliding*_ and *ε*_*G*-*sliding*_ were plotted as a function of M.A.R. The results show a linear relationship between this critical strain and M.A.R., as predicted by eq 1, providing a range of τ_*f*_ from 2.22 to 8.55 MPa. This shear strength value is comparable to the interactions between graphene–graphene layers in the literature (a very wide range of 0.04 MPa to 7 GPa, depending on different geometrical and microstructural factors^36−38^).

We plotted the *K*_*IC*_ of samples as a function of effective ductility. The details for toughness calculations are presented in Section S5. The graph, Figure 5(d), shows that as CNF length increases from 8 to 62 μm, the ductility increases by nearly 6 times, with a considerably lower decrease in *K*_*IC-eff*_ (∼30%). In other words, the considerable increase in critical displacement (and thus far field stress) is offset by the increase in the effective cross section area of the material with the addition of CNFs. Therefore, even though the addition of the CNFs postpones crack growth considerably, it has a limited impact on toughness. We would like to emphasize that the presence of CNFs does NOT increase the intrinsic ductility of graphene but, rather, makes it more resistant to crack growth by means of stress redistribution.

The calculated *K*_*IC-eff*_ for both neat SBLG and the hybrid material remains comparable although on the high side of reported values.^9,10,39,40^ The high toughness could reflect the nonlinear stress–strain relationship of graphene, which may lead to lower local stresses around crack tips compared to perfectly linear behavior.^8,10^ Another plausible explanation is rooted in the much larger size of our sample compared to most of the previous studies. When testing 2D materials with very small gage length (a few microns), the sample deformation remains purely 2D. In contrast, in our tests, the stretched sample may experience local buckling around the crack tip.^41^ We carried out a linear buckling analysis via FEA on the graphene to calculate the critical displacement for buckling to be ∼0.00318 nm, Figure S13(a). While the exact value of the critical buckling displacement remains unknown, it is far below the displacement at which cracks grow. Hence, the samples tested likely experience crack growth with local wrinkles. The buckled state around the crack tip may reduce stress singularity,^42,43^ requiring more far field stress to propagate the crack. However, the fracture toughness values reported here are with the assumption of 2D deformation, and as such, they are effective values reflecting the free-standing samples with allowable out-of-plane deformations. We would also like to point out that these mixed-dimensional materials retain a high surface-to-volume ratio with mass per unit area comparable to that of most 2D materials (see Section S2). Lastly, we calculated the probability of survival (*P*_*S*_ = ) for all the samples with/without the reinforcement of CNFs (details in Section S7).

In summary, incorporating CNFs between two graphene monolayers proves to be an effective approach for enhancing the load-bearing capacity of graphene. The CNFs contribute to crack bridging and deflection, making this composite material highly suitable for applications requiring high strength, toughness, and flexibility, especially for micro/nanostrain devices. The improved deformation capacity is mainly due to the placement location of the 1D nanofiller and their interactions. Molecular dynamics simulations reveal that tension has a significant effect on the profile conformity between the CNF and graphene layers, which was used to extract the tensile force applied in the experiment by comparing the profiles of the graphene layers under different tensile forces. During tensile testing, significant interfacial and axial sliding of the CNFs was observed, and the addition of CNFs led to considerable improvement in ductility and crack resistance, albeit at a slight loss in effective toughness. An elastic–perfectly plastic shear lag model was developed to estimate the true value of the shear strength and critical displacement required for interfacial sliding.

## Supporting Information Available

Schematic diagram of the synthesis and fabrication process, SEM image of the AFM tip, graphs of the AFM profile and least-square method, TEM images, MD simulations and graphs vs tension, Raman spectroscopy, in situ SEM images of all tensile testing, FEA simulations for stress distribution, schematic diagram for the shear lag model and its graph, and a graph of probability of survival with and without reinforcement (PDF)
