# Supplementary material for: Toughening Mechanisms in Stacked Bilayer Graphene Sheets by Means of Sandwiched 1D Nano-rebars
Source: Nano Lett. 2025 Apr 21;25(17):7153–60. doi: 10.1021/acs.nanolett.5c01513 (PMC12046602; doi:10.1021/acs.nanolett.5c01513)
Supplement: Supplementary file 1 — nl5c01513_si_001.pdf [file nl5c01513_si_001.pdf]

Supporting information file

Toughening Mechanisms in Stacked Bilayer Graphene Sheets by Means of Sandwiched 1D Nano-rebars

Muhammad Usama Arshad[%#], Yuxiang Gan[†#], Congjie Wei[†], Xingkang She[%], Pavan V. Kolluru[%], Chenglin Wu[†,*], Mohammad Naraghi [%,&,*]

#The two authors share the first authorship

[%]Department of Materials Science and Engineering, Texas A&M University, College Station, TX, 77843, USA.

[†]Department of Civil and Environmental Engineering, Texas A&M University, College Station, TX, 77843, USA.

[&]Department of Aerospace Engineering, Texas A&M University, College Station, TX, 77843, USA.

[*]Corresponding author, Email: Chenglin Wu chenglinwu@tamu.edu

[*]Corresponding author, Email: Mohammad Naraghi naraghi@tamu.edu

S1. Synthesis of CNF/SBLG composite

The step-by-step synthesis procedure of CNF/SBLG is demonstrated in **Figure S1 (a-h)**. In the first step, a large area monolayer graphene was grown on 2 × 10 cm copper foils using the chemical vapor deposition (Cu/Graphene). Carbon nanofibers (CNF), as the 1D reinforcements (rebar), made via pyrolysis of Polyacrylonitrile (PAN) nanofibers were drop casted on the monolayer graphene, and a thin layer of polymethyl methacrylate (PMMA) was spin-coated on Cu/Graphene/CNF at 3000 rpm for 30 seconds. The methods to produce CNFs can be found in an earlier work [1, 2]. The copper foil was then removed via etching by using a copper etchant for ~2 hours, and a free-standing graphene with CNF film was left behind supported by the PMMA layer (Gr/CNF/PMMA). For the fabrication of bilayer graphene, this Gr/CNF/PMMA film was first rinsed with deionized water and transferred onto another freshly synthesized monolayer graphene on copper foil. The sample was then heated at 50°C in dry air to eliminate any trapped water (Cu/CNF/SBLG/PMMA). The copper foil was again etched away to obtain the CNF/SBLG/PMMA composite film. Next, the CNF/SBLG/PMMA was transferred onto a TEM grid and heated to 120°C for 15 minutes to eliminate any remaining residues. PMMA was then removed by immersing the sample in an acetone solution for 1 hour. Any leftover polymer was further removed by etching at 400°C in a hydrogen atmosphere, resulting in a freestanding bilayer graphene film on the TEM grid. Each opening of TEM grid consists of a bilayer graphene with sandwiched CNFs. We chose TEM grids with one or two CNFs that are aligned with the loading direction (within 10°). To study crack growth in graphene with rebars, the TEM grids containing composite graphene samples were firmly mounted on a thin U-shaped aluminum wire using epoxy glue. To prepare the samples for fracture tests, focused ion beam (FIB) was used to cut and separate the sample from the TEM grid only along the two edges that are parallel to the far field loading,

and a crack was placed near the embedded CNF (within a few microns). The inset graphical image depicts the sample prepared for mechanical testing Figure S1 **(i-k)**.

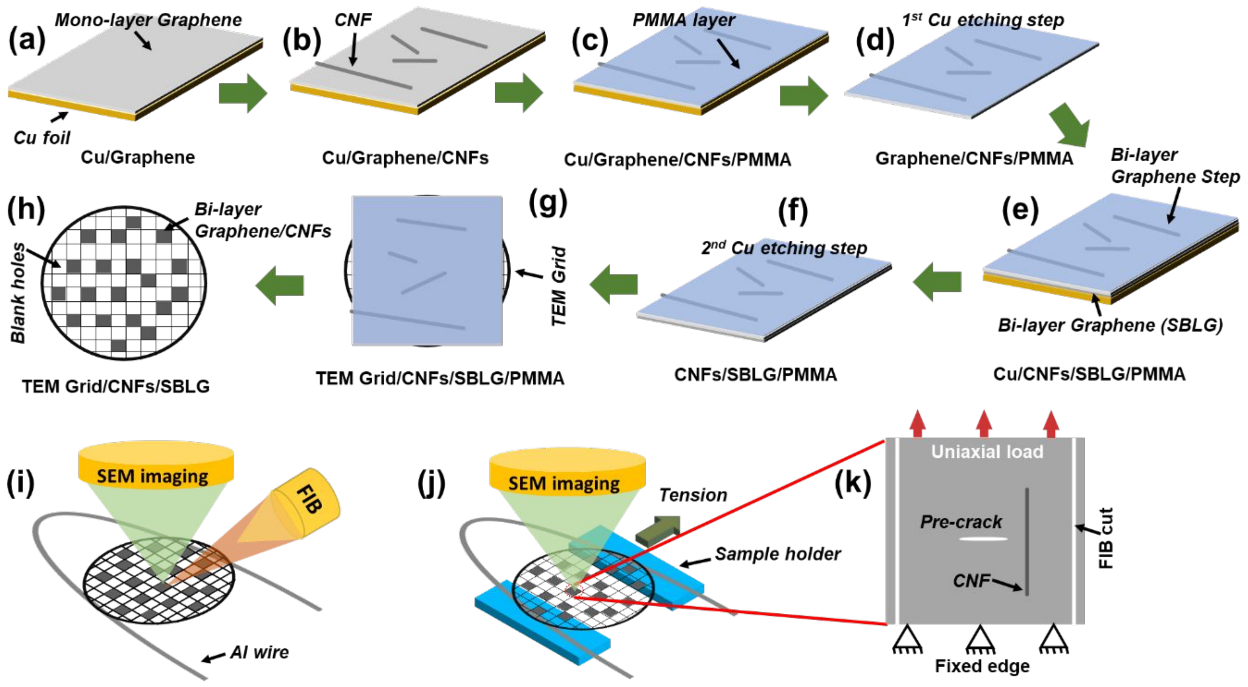

Figure S1. Schematic diagram of the complete synthesis and mechanical fabrication process.

S2. Geometric Conformity Analysis

To visualize the concept of geometrical conformity in CNF-Graphene structure, let's consider two flat horizontal sheets with negligible bending stiffness and a cylindrical prismatic bar held between them in space. The initial arrangement of these three bodies is such that the axis of the bar is parallel to the sheets, and it is equally spaced from (in between) the two sheets. We further assume that the three bodies have no initial overlap. This configuration is reminiscent of the two graphene sheets and CNFs prior to forming the sandwich structure. As the two sheets (i.e., graphene) are brought together to sandwich the cylindrical prismatic bar (i.e., CNF), at sufficiently far distances from the bar, the sheets will form a complete interface (full overlap) and the spacing between them vanishes, whereas in the proximity of the bar, the bar acts as a barrier for direct physical contact between the two. Assuming that the sheets are prevented from lateral sliding at their boundaries, achieving full conformity (i.e., graphene wrapping around CNFs) induces in plane stresses in graphene. Hence, conformity can be quantified as the angle at which the sheets are separated from the bar, measured from the apex of the bar in **Error! Reference source not found. (a)**. Therefore,

conformity will have a value of 0-90° where 0° conformity, nearly impossible to achieve, represents the case where the contact area between bar and sheets is reduced to a line, normal to the **Error! Reference source not found. (a)**, and a 90° conformity occurs when the sheets fully wrap the CNF.

S2.1. AFM Tip

Due to convolution of the imaged surface with the AFM tip geometry [3], the height profile obtained from AFM does not follow the exact geometry of the surface of graphene. In this section, geometrical arguments are presented to estimate the actual profile of graphene over CNF from the tip-convoluted AFM height profile. The tip geometry is shown in **Figure S2 (a)**. To estimate the actual height profile from the AFM height profiles which are convoluted by the AFM finite tip radius, we made the following assumptions:

- At the highest point of CNF (i.e., midsection of the AFM line scan), see Figure 2(d) of the main manuscript) graphene is in full contact with CNF, and the contact extends up to the separation angle θ. The separation point is shown with a red dot in Figure 2(a).
- Beyond the separation point and up until the two graphene sheets make full contact, the graphene has zero curvature (no bending) due to its insignificant bending stiffness.
- The AFM tip and graphene are mutually non-penetrable and rigid; thus, the geometrical approach laid out in [3] is used to deconvolute the effect of finite tip curvature.

Based on these assumptions, as the tip traverses along the line scans shown in **Figure 2(b)** of the main manuscript, the contact point between the tip and graphene can be identified mathematically as the point where the slopes of the tip/cone and the sample are equal [3], and the apparent AFM height profile can be tracked as the loci of AFM apex (i.e., the bottom most point of the AFM tip). It should be noted that from such an analysis, the top-most point of the height profile (relative to the far-field baseline) will always correspond to the height of the cylinder (i.e., radius of the CNF) on a flat plane[3]. In case of a CNF that is sandwiched symmetrically between two graphene layers, regardless of the extent of conformity, the highest point of the AFM height line scan represents the radius of the CNF. This is schematically illustrated in **Figure 2(c)** of the main manuscript where such an analysis of the AFM height profile indicates that the CNF imaged there had a diameter of ~360 nm diameter.

The aforementioned analysis was first applied to evaluate the expected AFM height profile for a graphene layer (relatively negligible thickness) that is fully conformal to a CNF with ~360 nm diameter. The AFM tip radius and half-cone angle required for this analysis were estimated via scanning electron microscopy (SEM) images, **Figure S2(a)**, to be ~18 nm and 16° respectively. The expected height profile (shown in Figure 2(d) of the main manuscript as a solid line), which accounts for tip convolution effects still does not match the experimentally measured profile (dashed line) and is in fact, always narrower than the experimental profile. This clearly indicates that the experimentally observed height profile cannot be due to tip convolution artifacts but is rather due to the non-conformal nature of graphene.

The above quantitative approach was also used to estimate the angle of separation, θ, to understand the extent or degree of non-conformity between graphene and CNF, and represents the angle between graphene and CNF at the point where graphene ceases to conform to the surface of CNF. We used the above assumptions to calculate the AFM profile for an arbitrary separation angle, and we used the least square method to find the separation angle at which the experimental and theoretical height profiles best match (minimizing the error). The error function was defined as:

$$Error = \sqrt{\frac{\sum_{k=1}^{n} \left(\mathrm{Y}_{Theory} - \mathrm{Y}_{Exp}\right)^2}{n}}$$

where Y_{Theory} and Y_{Exp} are respectively the height of the profile from theory (based on an arbitrary separation angle) and the experimental values at a particular horizontal location identified with integer variable k ranging from 1 to n.

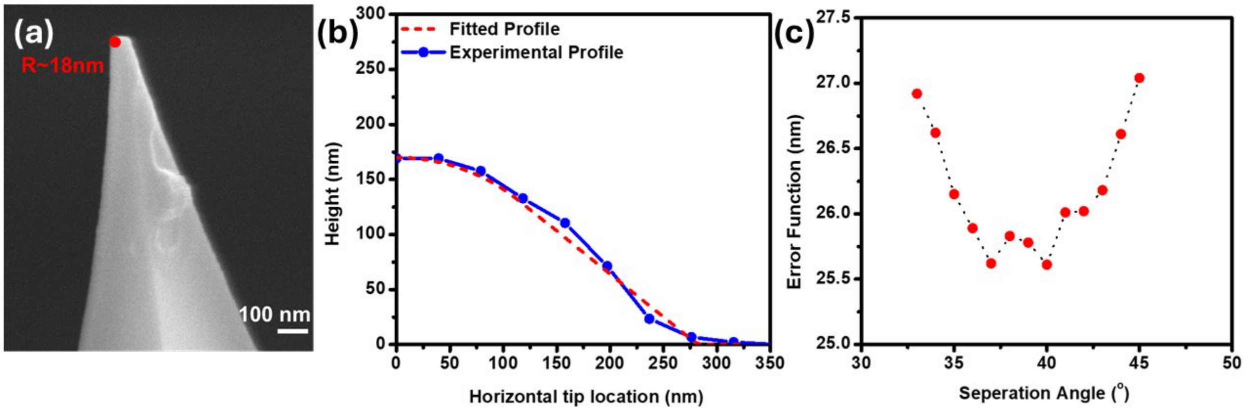

Figure S2. (a) SEM image of the AFM tip (b) Experimentally estimated AFM profile and fitted profile confirming the separating angle (c) Least square method to estimate the error function vs separation angle.

While the addition of CNFs extends the structure in the out-of-plane direction of graphene by half of the CNF diameter at the location of CNFs, the mass per unit area of the hybrid structure remains close to those of 2D materials. To demonstrate this trait, we calculated the mass per unit area of the material as m/A = $\frac{(\rho_G \times 2t \times A_G)+\frac{\pi}{4}D^2 \times \rho_{CNF} \times L_{CNF}}{A_G}$, where ρ_G is the density, t is the thickness, and A_G is the area of graphene, D is the diameter, ρ_{CNF} is the density and L_{CNF} is the length of CNF. We know ρ_{CNF} = 1.8 g/cm^3 and ρ_G = 2.26 g/cm^3. Thus, considering one of the samples for which $L_{CNF} = 8\ \mu m$, m/A= 1.94 g.nm/cm^3 = 1.94 mg/m^2, which is only ~26% higher than that of graphene. In the extreme case of the longest CNF studied which nearly spans the whole length of the graphene, $L_{CNF} = 62\ \mu m$, m/A= 4.74 g.nm/cm^3 = 4.74 mg/m^2. This is comparable to a six-layer graphene stack rather than a conventional thin film or coating in the 100–500 nm range.

S2.2. TEM Characterization

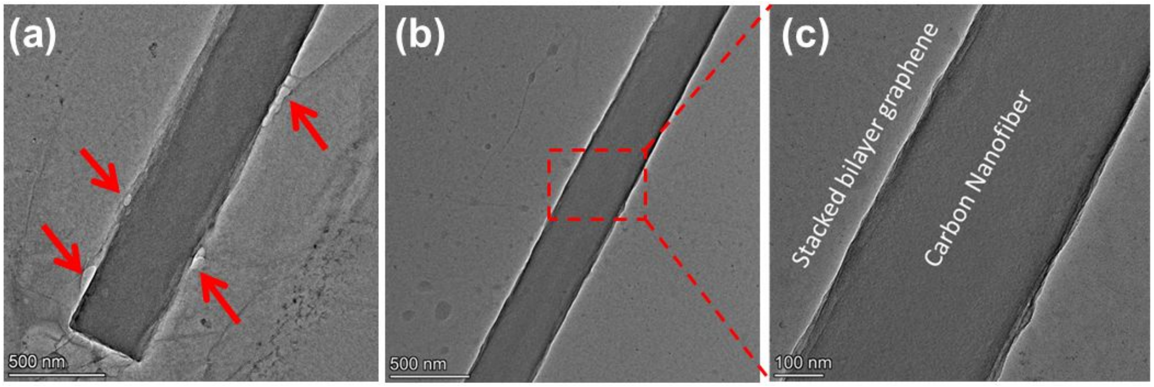

Figure S3. TEM image of the CNF/SBLG composite, (a) red arrows shows the defects at the interface, (b) shows the defect free CNF/SBLG composite interface (c) high magnification region for better clarity of the interface.

S2.3. Molecular Dynamic Simulations.

Two layers of graphene were simulated and positioned around the CNF. We allowed the graphene sheets to relax around the CNF, while being stretched by a variable in-plane stress in the direction normal to the CNF axis. This applied in-plane tension simulates a traction applied to the graphene by the sample holder during sandwiching the CNF and graphene while the graphene tends to wrap around CNF.

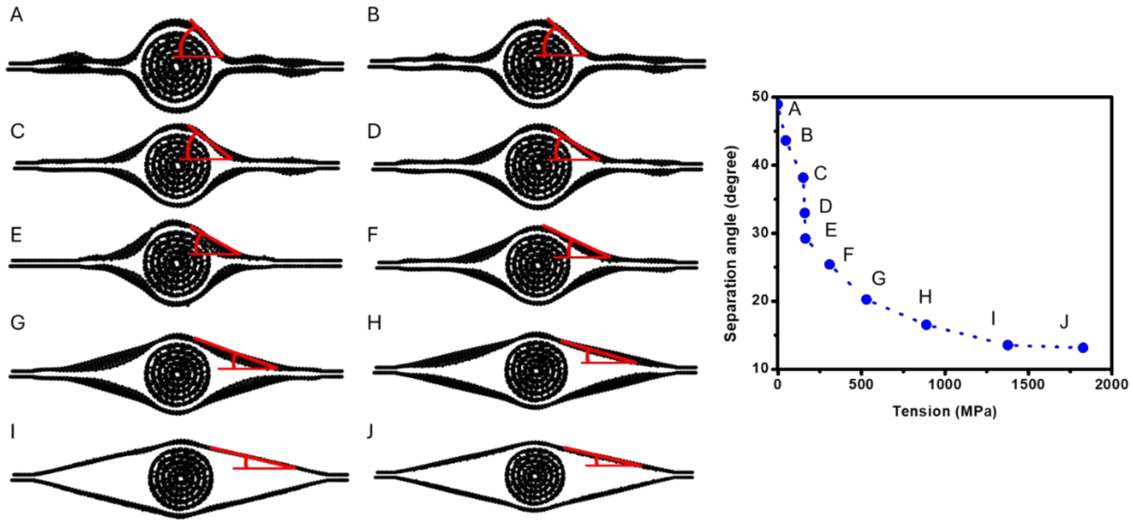

Figure S4. Simulations at different in-plane stresses in graphene, and the graph shows the conformal angle (separation angle) vs tension in the graphene sheet.

S2.4. Raman Spectroscopy Characterization.

Further insight into the morphology of graphene and CNF was obtained via Raman spectroscopy, **Figure S5**. Pristine SBLG exhibited an intense 2D peak at ~2637 cm⁻¹, which was considerably higher than that of the G peak at ~1590 cm⁻¹ (I_{2D}/I_G~1.629), indicating high crystallinity without any detectable D peak ~1345 cm⁻¹. However, CNFs displayed prominent D and G peaks at ~1345 cm⁻¹ and ~1590 cm⁻¹, respectively (I_D/I_G ~ 1.107), indicating a considerably high defect density without any 2D peak. Consequently, the Raman signal of CNF/SBLG exhibited the influence of CNFs, demonstrating a high I_D/I_G ~ 0.875.

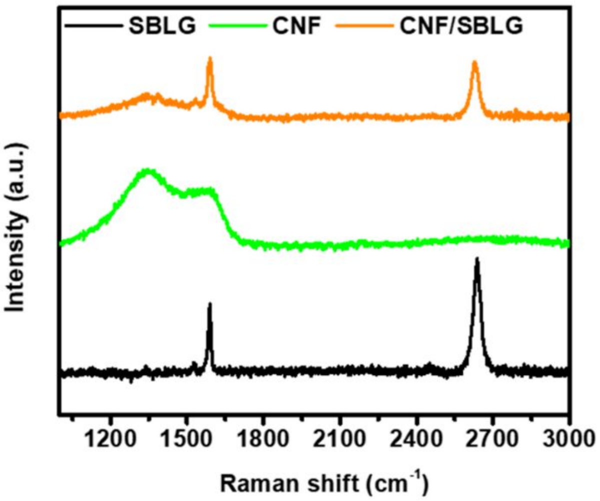

Figure S5: Raman spectroscopy of pristine SBLG, CNF mat and CNF/SBLG composite film.

S3. In-situ Tensile Testing of CNF/SBLG Composite

To measure δ_C, the TEM grid containing a few FIBed samples were stretched by applying a far field displacement in small increments, and after each increment of displacement, SEM images were taken to assess the average strain in the graphene and CNFs. The displacement was increased incrementally until the critical displacement required for crack growth was reached. The uniaxial load was incrementally applied to the TEM grid in the direction perpendicular to the initial crack (mode-I fracture) at a nominal strain rate of ~ 1.6×10^{-5} s^{-1}. The deformation was paused every ~ 60 seconds (corresponding to a nominal strain increment of ~10^{-3} or 0.1%) to capture both low and high magnification images of the samples. The uncertainty in the calculated displacement values was defined as the width of a single pixel of the SEM image. It is interesting to note that the critical average strain in graphene at which the CNF starts to slip scales linearly with the length of CNF. We will explain this relationship later by means of a shear lag model.

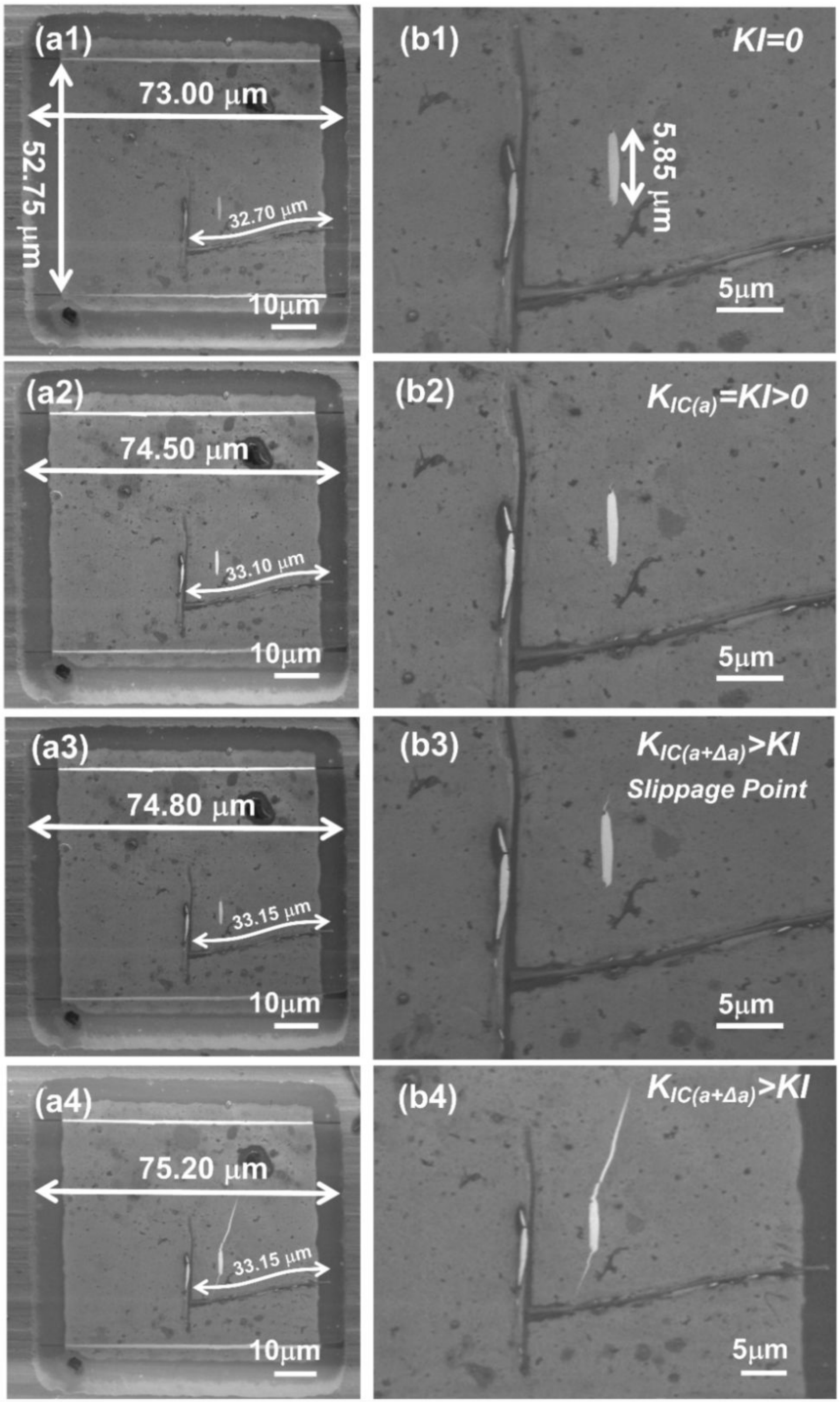

Figure S6. In-situ SEM images of tensile testing at different steps and magnifications, (a1-a4) before applying load, at the onset of crack propagation, at the onset of slippage and crack propagation, after further stable crack growth, respectively, (b1-b4) high magnification SEM images of the corresponding tensile test steps.

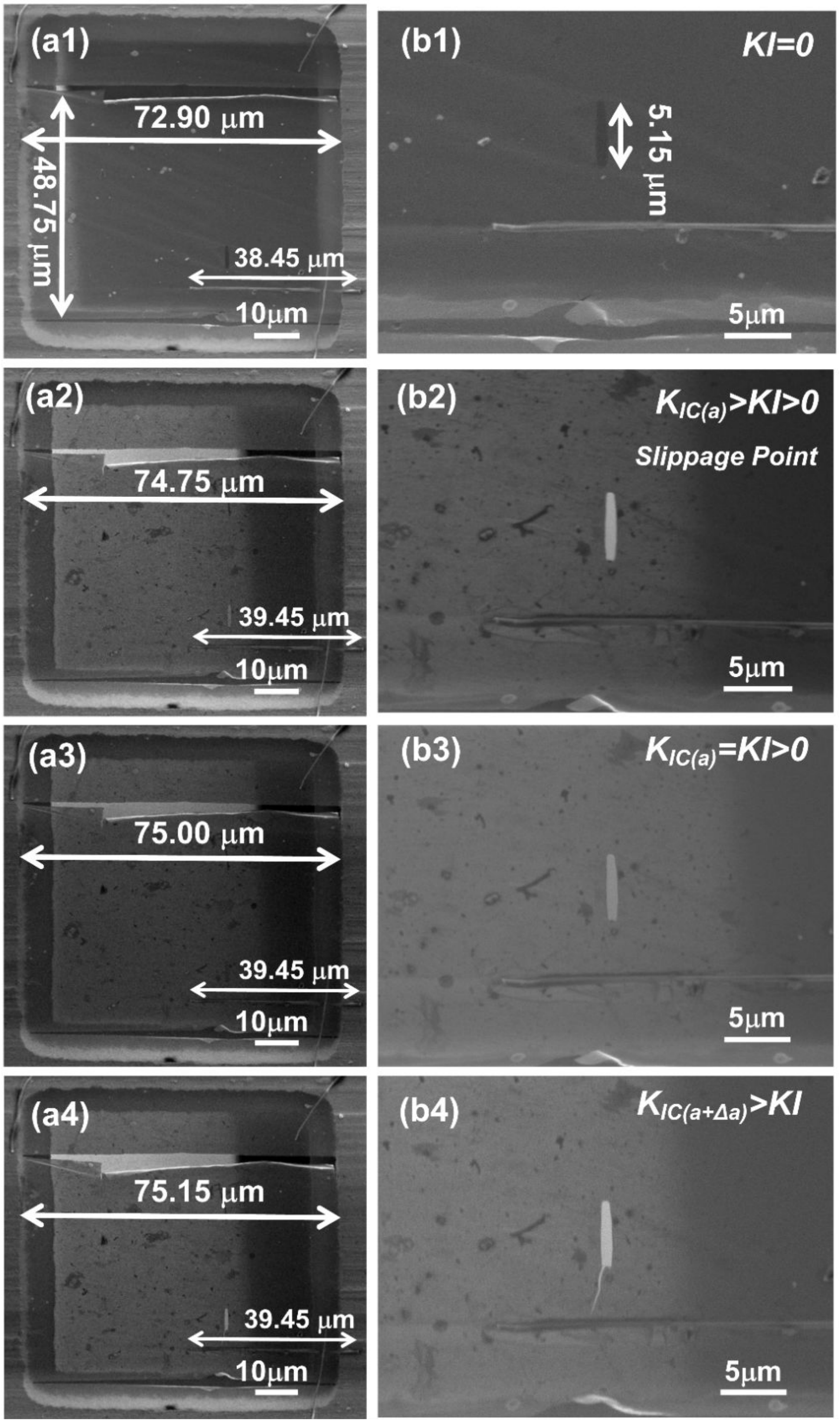

Figure S7. In-situ SEM images of tensile testing at different steps and magnifications, (a1-a4) before applying load, at the onset of slippage, at the onset of crack propagation, and after stable crack growth, respectively, (b1-b4) high magnification SEM images of the corresponding tensile test steps.

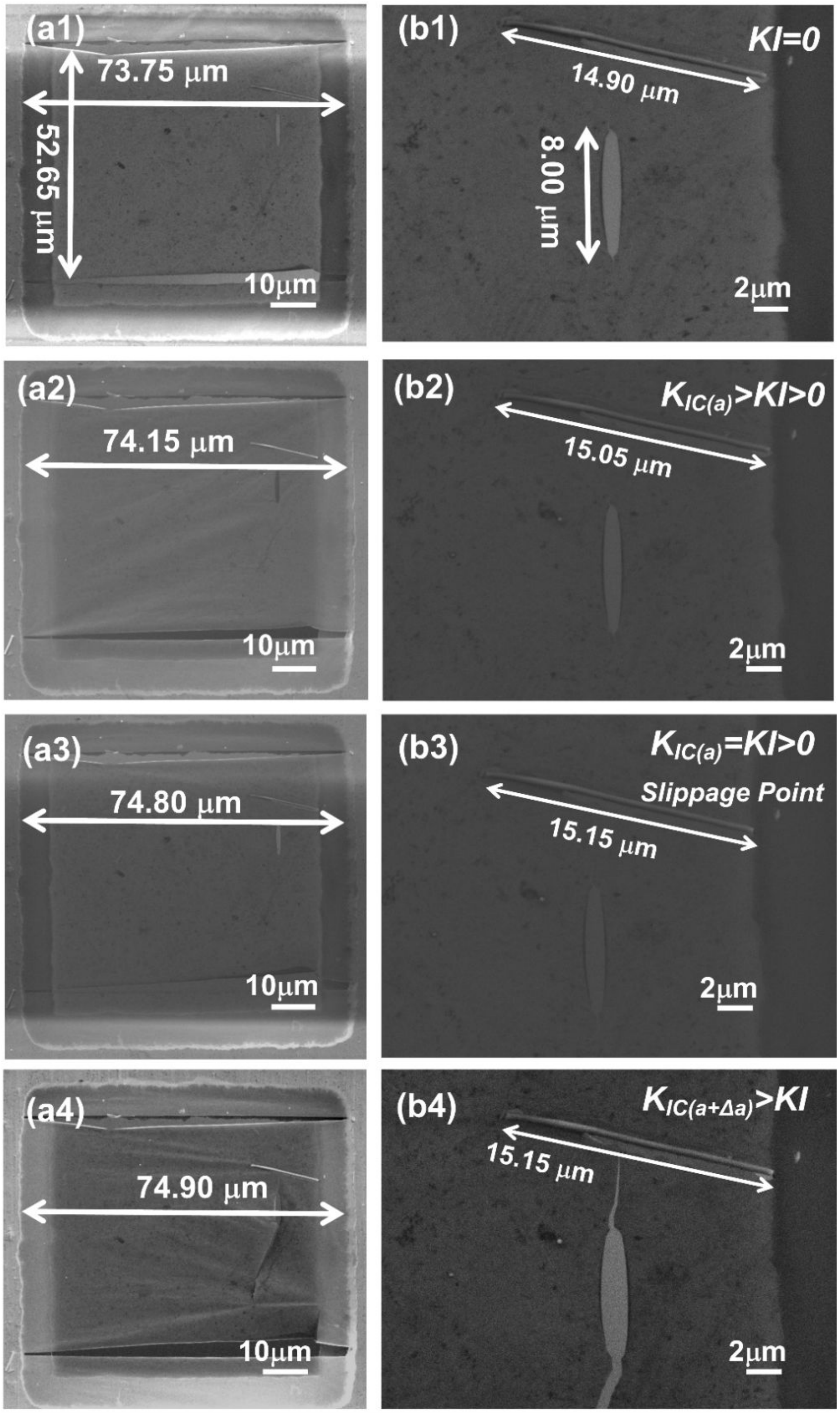

Figure S8. In-situ SEM images of tensile testing at different steps and magnifications, (a1-a4) before applying load, after applying some delta, at the onset of slippage and crack propagation, and after stable crack growth, respectively, (b1-b4) high magnification SEM images of the corresponding tensile test steps.

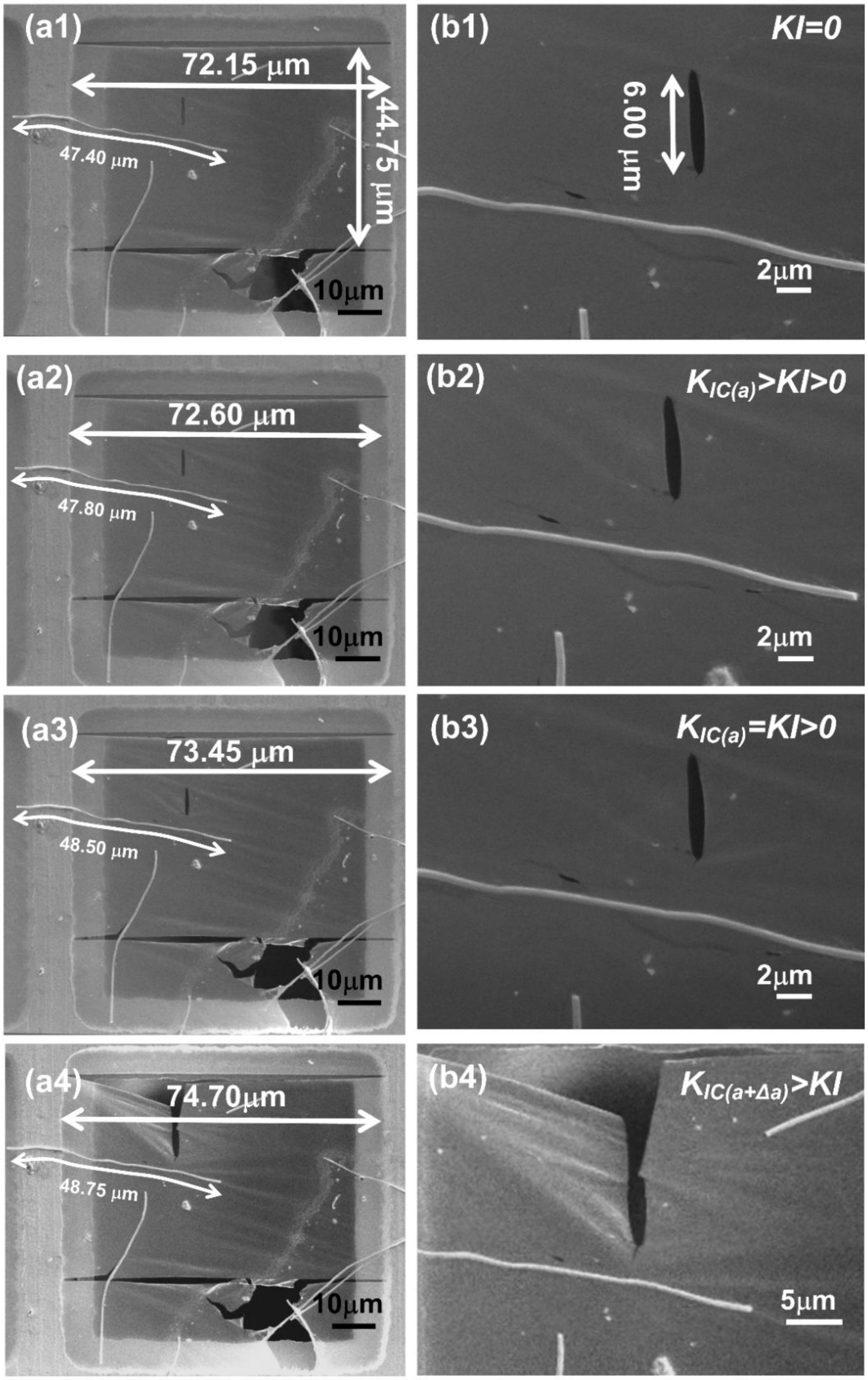

Figure S9. In-situ SEM images of tensile testing at different steps and magnifications, (a1-a4) before applying load, after applying some delta, at the onset of crack propagation, and after stable crack growth, respectively, (b1-b4) high magnification SEM images of the corresponding tensile test steps.

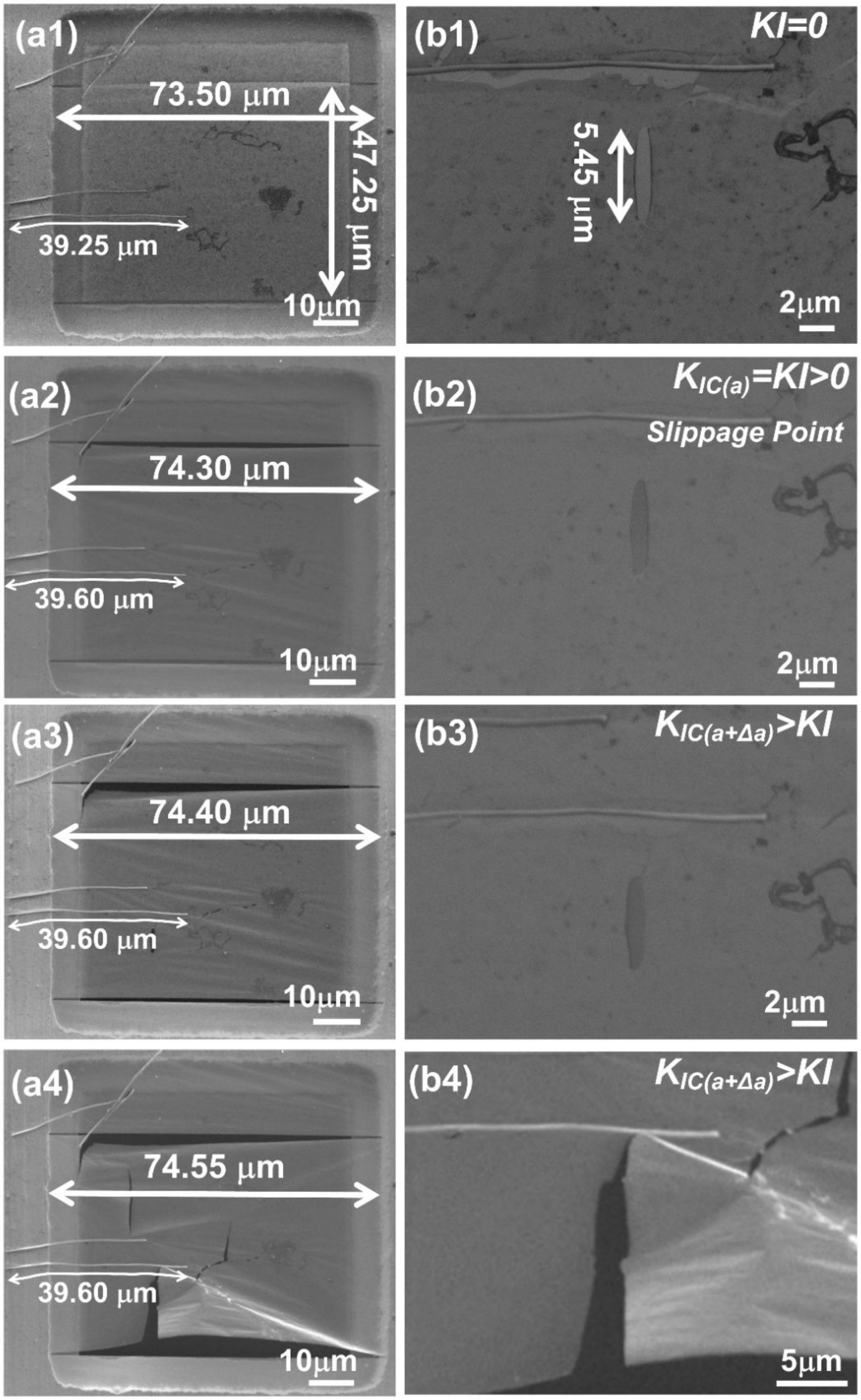

Figure S10. In-situ SEM images of tensile testing at different steps and magnifications, (a1-a4) before applying load, after applying some delta, at the onset of crack propagation, and after stable crack growth, respectively, (b1-b4) high magnification SEM images of the corresponding tensile test steps.

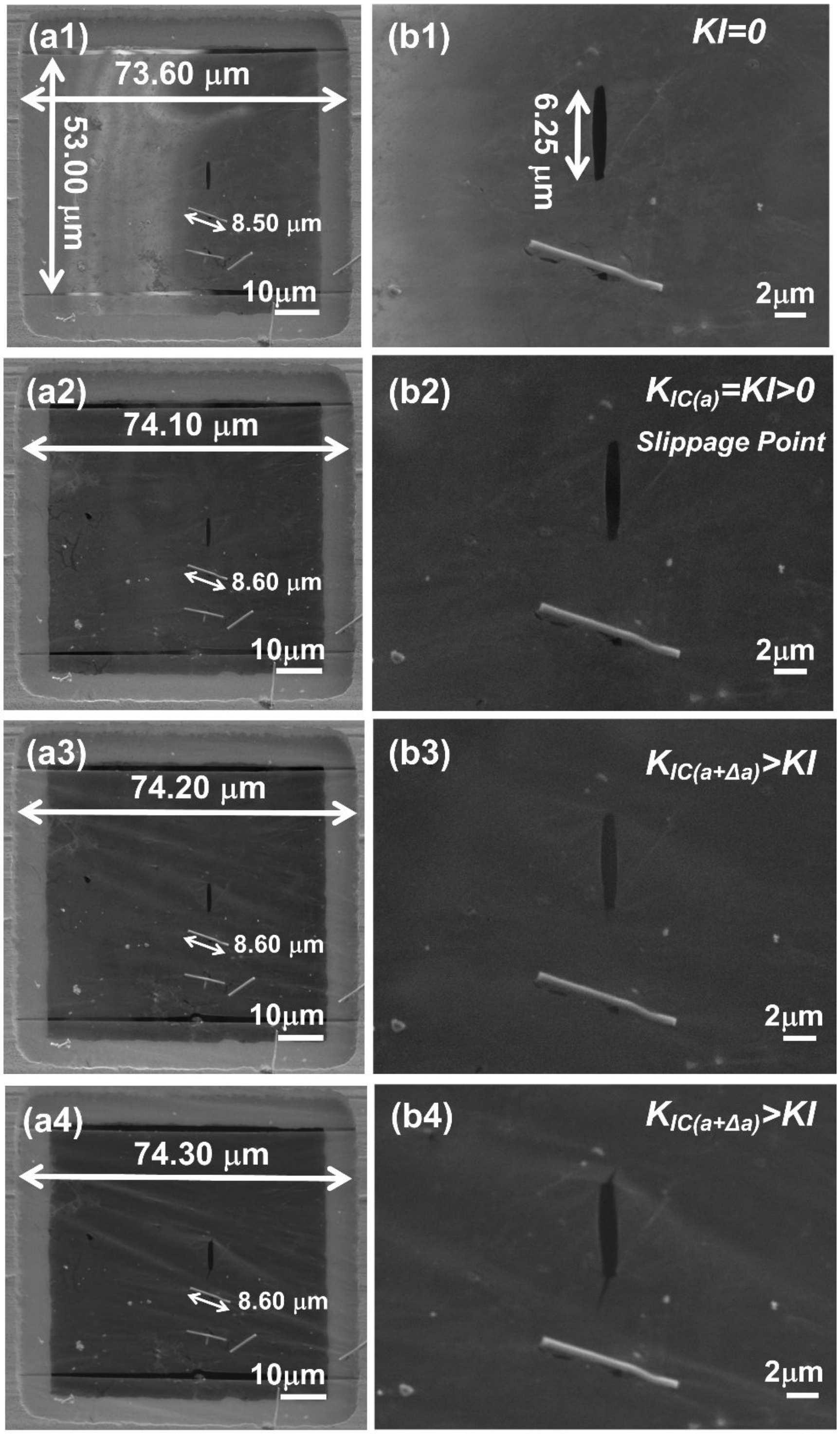

Figure S11. In-situ SEM images of tensile testing at different steps and magnifications, (a1-a4) before applying load, after applying some delta, at the onset of crack propagation, and after stable crack growth, respectively, (b1-b4) high magnification SEM images of the corresponding tensile test steps.

S4. Molecular Dynamics Simulations

Further insight about the load transfer between graphene and CNFs at the smaller scale was obtained via MD simulations (LAMMPS[4]) in which CNFs are pulled out of a graphene wrap. The ReaxFF potential was used to describe the interactions between atoms [5]. As shown in **Error! Reference source not found.(a)**, a graphene-CNF-graphene composite structure is first constructed. The graphene layers are positioned on the CNF along the top and bottom surfaces, conforming to their natural contours. Along the direction perpendicular to the CNF the graphene layers were given a width sufficient to clearly display the transition from the CNF-graphene composite to double layer graphene structure unaffected by the presence of CNFs, shown as the sections colored with blue in **Error! Reference source not found.(a)**. This structure is first relaxed without any applied load at both ends. Two interaction scenarios were considered between the CNF and graphene layers on both sides: fully bonded, where the top and bottom graphene sections were bonded with CNF and move together, and [4] weak interaction via Van der Waals forces, where only the CNF is moved while the graphene sections were set. The latter represents the experiments presented in this work, and the consideration of the strong interface is intended to provide more insights into the effect of interface on mechanics. For both cases, the graphene structures of both sides were fixed to have no displacements along all directions while the CNF is moved along out-of-plane direction at a constant speed and has no deformation. The modeling results are shown as the right subfigure of **Error! Reference source not found.(a)**. For a clear illustration, only a quarter of the whole structure is used to develop these figures. From these results, both interfaces have developed wrinkles caused by the pulling out of the CNF. However, for the bonded case, the graphene layers were ripped, and a crack was developed in the graphene layer, located between the bonded and the transition sections. Correspondingly, several wrinkles with large amplitudes were also observed in this case and extended all the way to the double layer graphene boundaries. As for the cases with only Van der Waals interaction, the number of wrinkles increased but the amplitude is greatly decreased. The graphene sections on top of the CNF have a much smaller deformation along the direction of the CNF movements. This is consistent with the strain distributions for both cases shown in **Error! Reference source not found.(b)**. To conduct a quantitative analysis, first, we determined the wrinkles caused by the interaction between the CNF and graphene rather than the natural wrinkles commonly observed in the free-standing graphene structures. According to the work on[6], the natural wrinkles in graphene have an

amplitude over wavelength ratio around 1/50, which is set as the threshold to distinguish the interaction-caused wrinkles (i.e., wrinkles with higher amplitude are attributed to the applied load and type of interactions). Furthermore, the profile of graphene transition sections on the representative plane were extracted and shown in **Error! Reference source not found.(c)**. Through analysis of the shape and locations, the domain containing these wrinkles is defined as the cohesive zone while the lengths for both cases are calculated and shown as $l_{coh,bond}$ and $l_{coh,vdw}$ in **Error! Reference source not found.(c-d)**. The cohesive zone for the bonded case covers the whole transition domain while that for the Van der Waals case covers 84%. Given the high distortion of graphene in the bonded case, it seems reasonable to assume that bonded interactions in a loaded 1D-2D structure may increase the stress in graphene, leading to graphene failure near the interface, as a prelude for more catastrophic failures.

S5. Finite Element Analysis Simulations and Estimation of Effective Toughness

In the FEA phase field analysis, the elastic modulus of graphene and CNF were considered 1 TPa and 80 GPa. While our in-situ mechanical experiments focused on measuring elongation and its critical value at crack initiation, we leveraged models to estimate the applied force at this onset. Specifically, phase field finite element (FE) simulations were conducted to determine the far-field stress at crack initiation, incorporating experimentally measured mechanical properties of SBLG and CNFs from previous studies[7, 8]. These simulations reveal crack initiation, bridging mechanisms (see Section S5), and stress distribution, highlighting the toughening effect of CNFs. Additionally, the model provided the critical load, $F_{C,}$ applied at the graphene boundary at crack onset, from which the effective fracture toughness of the SBLG-CNF system was calculated as: $K_{IC-eff} = Y\sigma_{\infty}\sqrt{\pi a}$, where $\sigma_{\infty}$is the average far field stress applied at the boundaries at the onset of crack growth, $\sigma_{\infty} \sim \frac{F_C}{A_{eq}}$. Here, $A_{eq}$is the equivalent cross-sectional area of the SBLG-CNF system calculated in each sample as the ratio of the total volume of CNF and SBLG (thickness times the length of graphene) divided by graphene length along the loading direction.

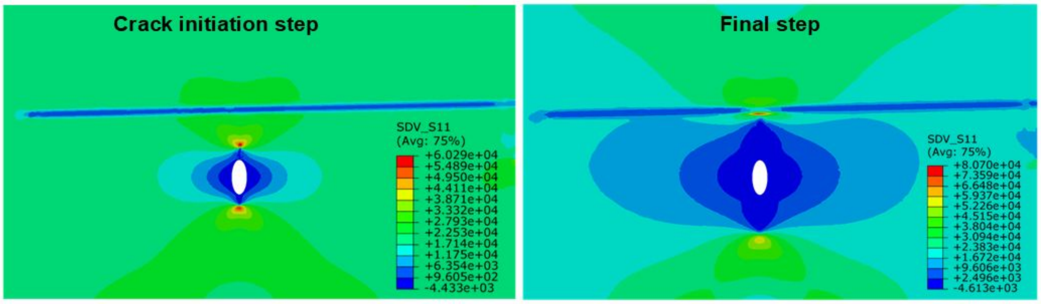

Figure S12: Finite element Phase field modeling, shows the stress distribution at two different steps.

During our in-situ experiments, we observed indications of local buckling (such as changes in the sample contrast, reminiscent of out of plane deformation around the crack tip, even though it cannot be quantified), encouraging us to conduct Linear Elastic Fracture Mechanics (LEFM) simulations to visualize the buckling and estimate the responsible onset critical displacement Figure S13. The analysis exhibited that the critical displacement causing buckling is ~0.00318 nm Figure S13(a), which is far below any experimentally measurable quantity. In contrast, the average critical displacement for crack growth in SBLG was calculated to be ~0.65 μm. This finding indicates that, due to the nanometer-scale thickness of graphene, the buckling near the pre-crack occurs significantly earlier than crack growth. We verified our buckling model with Rammerstorfer model[9].

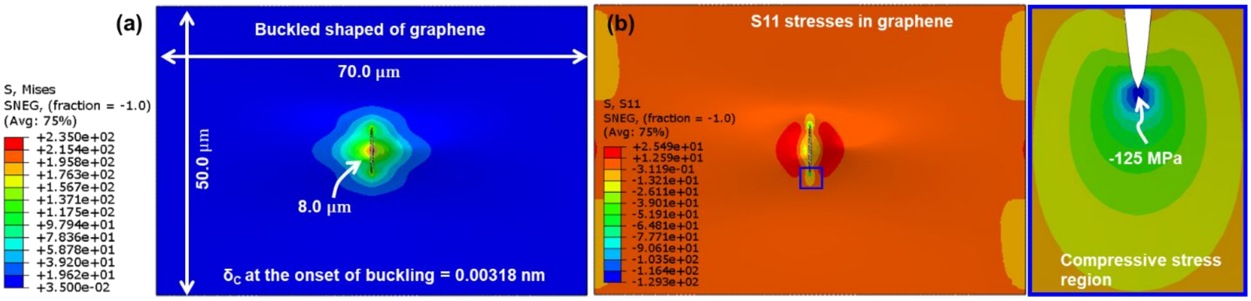

Figure S13: (a) FEA simulations for the visualization of buckling and the estimation of critical displacement at the onset of buckling (b) stresses in x-direction (S11) for the visualization and estimation of compressive stresses

S6. Shear Lag Model

S6.1. Mechanics of CNF/SBLG composite film

Fully elastic CNF-graphene interface:

Consider a single CNF with length L that is sandwiched between two graphene sheets. The geometry is schematically shown in Figure S11. The graphene in this model is the SBLG. The graphene and CNF were assumed to be linear elastic materials, with elastic moduli of 80 GPa and 1 TPa, respectively. The elastic modulus of graphene was obtained from the push-to-pull microdevice experiments, as reported in [10], and the elastic modulus of the CNF was obtained from microdevice experiments, as reported in[8]. The SBLG is stretched on both ends by a force of $\sigma_0 wt$ in the direction along the fiber axis. The CNF is semi-continuous in this problem; it starts at x = 0, and continues beyond x = L. This load is entirely carried by the graphene at the x = 0 (one end of the CNF), while the force is shared between CNF and graphene at x = L.

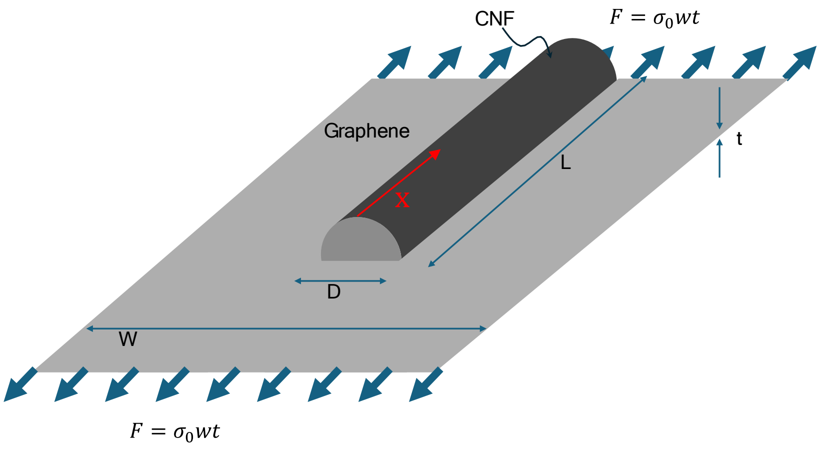

Figure S14. *The schematic of the geometrical parameters used to develop the shear lag model.*

Moreover, the CNF is sandwiched in between the two individual layers of graphene; the load transfer between them is via van der Waals forces. As it is commonly carried out in shear lag models, the interface is modeled as a continuum with elastic or elastic-perfectly plastic behavior (See Figure 5b of the manuscript). The shear modulus is *G*, thickness of the interface is *t* and it yields at shear stress of τ_f [11, 12]. For a unit length, the force equilibrium balance yields:

$$\tau(x) = \frac{4\chi}{D}\frac{d\sigma_C(x)}{dx}$$ ***Eq. S1***

$$\tau(x) = -\frac{\chi\pi D}{wt}\frac{d\sigma_G(x)}{dx}$$

where $\tau(x)$ is the shear stress distribution along the interface, $\sigma_C(x)$and $\sigma_G(x)$ are the axial stresses in the CNF and graphene, respectively. Moreover, $\chi = \frac{\theta}{\pi/2}$ is the fraction of the circumference of the CNF that is in contact with CNF, with θ being the separation angle (main manuscript, Figure 2).

First, we will assume that the applied stress is sufficiently low such that the interface remains elastic:

$$\tau(x) = G\gamma(x) \quad \textit{Eq. S2}$$

Differentiate on both side w.r.t "x", we get:

$$\frac{d\tau(x)}{dx} = G\frac{d\gamma(x)}{dx} \quad \textit{Eq. S3}$$

Moreover, the interface shear strain can be presented as the difference between the axial displacement of CNF and graphene divided by the gap between them, or $\gamma(x)$=$\frac{U_C(x)-U_G(x)}{h}$. Therefore, the change in the shear strain $\gamma(x)$ can be written as:

$$\frac{d\gamma(x)}{dx} = \left[\frac{\frac{dU_C(x)}{dx} - \frac{dU_G(x)}{dx}}{h}\right] \quad \textit{Eq. S4}$$

Substituting **Eq. S4** into **Eq. S5**, we get:

$$\frac{\mathrm{d}\tau(x)}{\mathrm{d}x} = \frac{G}{h}\left[\frac{\sigma_C(x)}{E_C} - \frac{\sigma_G(x)}{E_G}\right] \quad \textit{Eq. S5}$$

Differentiate **Eq. S1** on both side w.r.t "x", we get:

$$\frac{d^2\sigma_C}{dx^2} = \frac{4\chi}{D}\frac{\mathrm{d}\tau(x)}{\mathrm{d}x} \quad \textit{Eq. S6}$$

$$\frac{d^2\sigma_G}{dx^2} = -\frac{\chi\pi D}{wt}\frac{\mathrm{d}\tau(x)}{\mathrm{d}x}$$

Substitute **Eq. S5** into **Eq. S6**.

$$\frac{d^2\sigma_C}{dx^2} = \frac{4\chi G}{Dh}\left[\frac{\sigma_C(x)}{E_C} - \frac{\sigma_G(x)}{E_G}\right]$$

$$\frac{d^2\sigma_G}{dx^2} = \frac{\chi\pi DG}{wth}\left[\frac{\sigma_G(x)}{E_G} - \frac{\sigma_C(x)}{E_C}\right]$$

Eq. S7

To simplify the above equations, we defined new parameters:

$\lambda^2 = \frac{G}{E_C h b_1}$; $n = \frac{E_C}{E_G}$; $b_1 = \frac{D}{4\chi}$; $b_2 = \frac{wt}{\chi\pi D}$; $m = \frac{b_1}{b_2} = \frac{\pi D^2}{4wt}$; $k^2 = mn + 1$

Hence, **Eq. S7** can be written as:

$$\frac{d^2\sigma_C}{dx^2} = \lambda^2[\sigma_C - n\sigma_G]$$

$$\frac{d^2\sigma_G}{dx^2} = m\lambda^2[n\sigma_G - \sigma_C]$$

Eq. S8

Boundary Conditions (BCs):

At $x = 0$ $\sigma_C(0) = 0$; $\sigma_G(0) = \sigma_0$

At $x = L$ $\sigma_C(L) = \frac{n\,\sigma_0}{k^2}$; $\sigma_G(L) = \frac{\sigma_0}{k^2}$

The above set of BCs are obtained by the following considerations. The CNF does not continue past the x = 0, therefore, it does not carry any load, and all the load is carried by the CNF. On the other end at x = L, the total amount of load will be shared between CNF and graphene. To achieve the BCs at this end, we have assumed that the overlap length between the CNF and graphene is sufficiently long such that they reach the same strain (shear stress approaches zero). Hence, two conditions are met at x = L. First, the ratio of the stresses in graphene and CNF is the same as the

ratio of their elastic moduli. Second, the sum of the forces carried by CNF and graphene should be the same as $\sigma_0 wt$ to maintain the equilibrium of the whole structure. From these two conditions, the BCs at x = L can be obtained. The closed form solution of the above ordinary differential equations with these BCs is:

$$\sigma_C = \frac{n\,\sigma_0}{k^2}\left[1 - \frac{\sinh(\lambda k(L-x))}{\sinh(\lambda kL)}\right] \quad \textit{Eq. S9}$$

$$\sigma_G = \frac{\sigma_0}{k^2}\left[\frac{\sinh(\lambda kL) + (k^2-1).\,(\sinh(\lambda k(L-x)))}{\sinh(\lambda kL)}\right] \quad \textit{Eq. S10}$$

Using the above equations the shear stress distribution over the interface can be written as:

$$\tau(x) = \frac{\lambda b_1 n \sigma_0}{k}\left[\frac{\cosh(\lambda k(L-x))}{\sinh(\lambda kL)}\right] \quad \textit{Eq. S11}$$

Fully plastic CNF-graphene interface:

According to Eq. 11, by increasing the applied stress (and deformation), shear stress will increase monotonically and proportionally everywhere along the interface. This trend continues until the shear stress at x = 0 (maximum of $\tau(x)$ for elastic interface), $\frac{\lambda b_1 n \sigma_0}{k}\left[\frac{\cosh(\lambda k\mathrm{L})}{\sinh(\lambda kL)}\right]$, reaches the shear strength τ_f (Elastic-perfectly plastic interface model). That is, the interface will start to deform plastically at x = 0. Further increasing the applied deformation and far field stress σ_0 will not change the value of shear stress at x = 0, as it has reached its material limit. Instead, the size of the plastic zone will increase until the whole interface becomes plastic. The spread of the plastic zone along the whole interface leads to a noticeable sliding of the CNF in the experiment. For the investigation of shear stress when the plastic region has spread all along the interface, we started with the same initial balance of forces equation (Eq. S1) with the assumption that $\tau_{(x)} = \tau_f$:

$$\frac{d\sigma_C}{dx} = \frac{4\chi}{D}\tau_f$$

$$\frac{d\sigma_G}{dx} = -\frac{\chi\pi D}{wt}\tau_f \quad \textit{Eq. S12}$$

Integrate w.r.t "x":

$$\sigma_C = \frac{4\chi}{D}\tau_f.x + C_1$$

Eq. S13

$$\sigma_G = -\frac{\chi\pi D}{wt}\tau_f.x + C_2$$

The BCs remain the same:

At $x = 0$ $\sigma_C(0) = 0$; $\sigma_G(0) = \sigma_0$

At $x = L$ $\sigma_C(L) = \frac{n\,\sigma_0}{k^2}$; $\sigma_G(L) = \frac{\sigma_0}{k^2}$

Hence, at $x = 0$:

$$\sigma_C = \frac{4\chi}{D}\tau_f.x$$

Eq. S14

$$\sigma_G = -\frac{\chi\pi D}{wt}\tau_f.x + \sigma_0$$

And at $x = L$

$$\frac{n\,\sigma_0}{k^2} = \frac{4\chi}{D}\tau_f.L$$

$$\sigma_G = -\frac{\chi\pi D}{wt}\tau_f.L + \sigma_0$$

Eq. S15

$$\frac{\sigma_0}{k^2} = -\frac{\chi\pi D}{wt}\tau_f.L + \sigma_0$$

We can rewrite **Eq. S14** in terms of strain of graphene by dividing it by the elastic modulus:

$$\varepsilon_G = -\frac{\chi\pi D}{wtE_G}\tau_f.x + \frac{\sigma_0}{E_G}$$

Eq. S16

Integrate **Eq. S16** over the total length of graphene and express interns of δ_C:

$$\delta_C = -\frac{\chi\pi D}{wtE_G}\tau_f.\frac{L^2}{2} + \frac{\sigma_0}{E_G}L_{total}$$

Eq. S17

Here, "L" is the length of CNF and "L_{total}" is the total length of graphene (overlapped length plus remaining length of graphene).

We can rewrite the **Eq. S17** for far field stress (σ_0).

$$\sigma_0 = \frac{\delta_C E_G}{\left[L_{total} - \frac{n\pi D^2 L}{8wtK^2}\right]} \qquad \textit{Eq. S18}$$

Hence, substituting the value of σ_0 in ***Eq. S15*** and solve further for shear stress τ_f, we get:

$$\tau_f = \frac{n\sigma_0 D}{4\chi k^2 L} \qquad \textit{Eq. S19}$$

Between **Eq. S18** and **S. 19**, we can eliminate σ_0 and obtain a simple relationship between the interface shear strength and applied displacement at the onset of sliding (**Figure S12**).

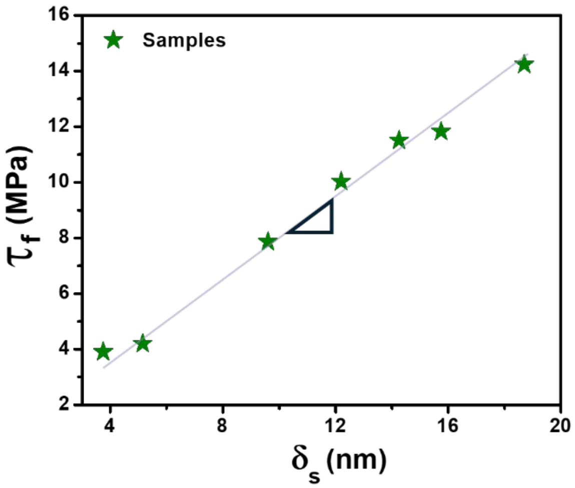

Figure S15: Relationship between true τ_f vs δ_s. Green stars exhibiting different samples. The value of δ_C is obtained from experiments, and the τ_f is calculated from Eq. S18 & S19.

S6.2. Dependence of load bearing capacity of graphene on length of CNF

The combination of Eq. S18 and S.19 will provide us with a relationship between the cirtical applied displcaement and interfacial shear strength which is nonlinear in terms of the CNF length. An approximation of the relationship between critical displacement at the onset of sliding and itnerfacial shear strength can also be obtained here in which the second order terms are ignored. This analysis is based on the assumption that the stiffness of the CNF is much smaller than that of graphene, and as such the changes in the axial stress in grapehene along the x axis is approximated

as being linear. Therefore, applying the BCs of stress for graphene, the average strain in graphene will:

$$\sigma_G \cong \frac{\sigma_0}{2}\left(1+\frac{1}{k^2}\right) = E_G \varepsilon_G \qquad \textit{Eq. S20}$$

A shear stress component over the length of CNF is described as follows (the change in axial force of graphene between x = 0 and x = L should be carried by the CNF through the interface):

$$\tau_f \chi \pi D L_C = \left(\sigma_0 - \frac{\sigma_0}{k^2}\right) wt \qquad \textit{Eq. S21}$$

Divide the **Eq. S20** with **Eq. S21** and we get:

$$\frac{\tau_f \chi \pi D L_C}{E_G \varepsilon_G} = \frac{\sigma_0\left(1-\frac{1}{k^2}\right)wt}{\frac{\sigma_0}{2}\left(1+\frac{1}{k^2}\right)} \qquad \textit{Eq. S22}$$

$$\frac{\tau_f \chi \pi D L_C}{2 E_G \varepsilon_G wt} = \frac{\left(1-\frac{1}{k^2}\right)}{\left(1+\frac{1}{k^2}\right)} \qquad \textit{Eq. S23}$$

Rearranging the equation **Eq. S23** for ε_G gives a linear relationship of critical strain in CNF and graphene at the onset of sliding with the length of CNF:

$$\varepsilon_{CNF-sliding} = \varepsilon_{G-sliding} = \left(\frac{1+\frac{1}{k^2}}{1-\frac{1}{k^2}}\right) \times \frac{\tau_f \chi \pi D}{2wtE_G} L_{CNF} \qquad \textit{Eq. S24}$$

Or

$$\varepsilon_{CNF-sliding} = \varepsilon_{G-sliding} = \overline{\tau_f}\beta$$

Where,

$$\overline{\tau_f} = \tau_f / \left(\frac{2tE_G}{\chi \pi D}\right) \text{ and } \beta = \frac{L_{CNF}}{w} \times \left(\frac{1+\frac{1}{k^2}}{1-\frac{1}{k^2}}\right).$$

Here, $\overline{\tau_f}$ is a non-dimentionalized shear strength defined as the ratio of shear strength over $\frac{2tE_G}{\chi\pi D}$, which is the same for all samples, while the value of β varies from sample to sample.

S7. Probability of Survival.

Data set of the SBLG without the reinforcement of CNFs were taken from our previous study [10]. All samples were ranked based on the critical displacement required for crack propagation (δ_C) from lowest to highest, and the probability of survival (P_S) for both the SBLG and CNF/SBLG composite was estimated as $P_S = 1 - (\frac{i-0.5}{N})$, where "i" represents the rank order of the specific sample, and "N" is the total number of samples. For *SBLG*, the sample with the lowest δ_C (0.3 µm) had a rank of 1, resulting in a survival probability of approximately 92.85% at δ_C = 0.3 µm. Conversely, the sample with the highest δ_C (0.9 µm) had a rank of N (7), leading to a survival probability of approximately 7.14% at δ_C = 0.9 µm. Similarly, for the CNF/SBLG composite, the sample with the lowest δ_C (0.6 µm) had a rank of 1, yielding a survival probability of about 92.85% at δ_C = 0.6 µm, while the sample with the highest δ_C (3.05 µm) had a rank of N (7), resulting in a survival probability of approximately 7.14% at δ_C =3.05 µm.

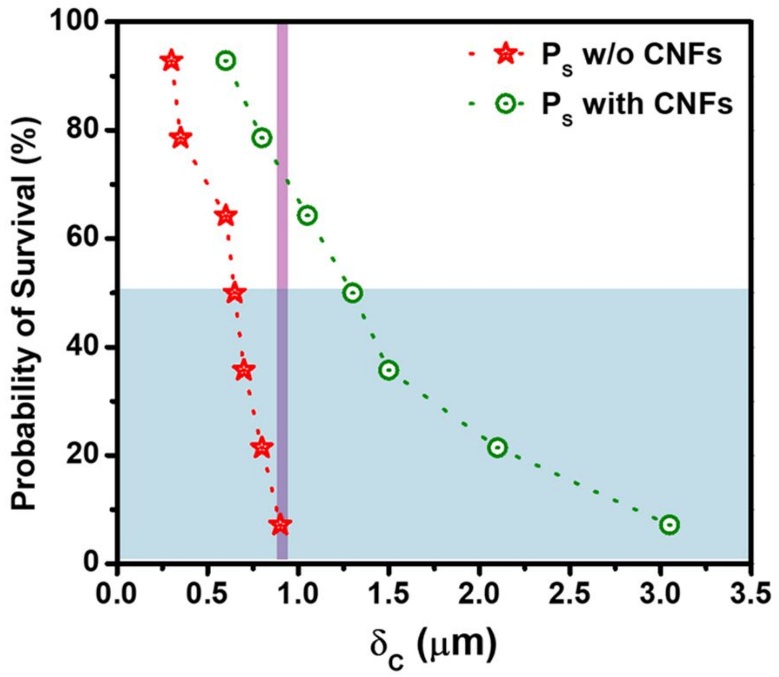

Figure S16: Probability of survival vs critical displacement with and without the reinforcement of CNFs

References

1. Chawla, S.; Cai, J. Z.; Naraghi, M., Mechanical tests on individual carbon nanofibers reveals the strong effect of graphitic alignment achieved via precursor hot-drawing. *Carbon* **2017,** *117*, 208-219.

2. Cai, J.; Naraghi, M., Non-intertwined graphitic domains leads to super strong and tough continuous 1D nanostructures. *Carbon* **2018,** *137*, 242-251.

3. Odin, C.; Aimé, J. P.; El Kaakour, Z.; Bouhacina, T., Tip's finite size effects on atomic force microscopy in the contact mode: simple geometrical considerations for rapid estimation of apex radius and tip angle based on the study of polystyrene latex balls. *Surface Science* **1994,** *317* (3), 321-340.

4. Kim, S.-Y.; Kumar, N.; Persson, P.; Sofo, J.; Van Duin, A. C.; Kubicki, J. D., Development of a ReaxFF reactive force field for titanium dioxide/water systems. *Langmuir* **2013,** *29* (25), 7838-7846.

5. Kim, S.-Y.; Kumar, N.; Persson, P.; Sofo, J.; van Duin, A. C. T.; Kubicki, J. D., Development of a ReaxFF Reactive Force Field for Titanium Dioxide/Water Systems. *Langmuir* **2013,** *29* (25), 7838-7846.

6. Nicholl, R. J. T.; Conley, H. J.; Lavrik, N. V.; Vlassiouk, I.; Puzyrev, Y. S.; Sreenivas, V. P.; Pantelides, S. T.; Bolotin, K. I., The effect of intrinsic crumpling on the mechanics of free-standing graphene. *Nature Communications* **2015,** *6* (1), 8789.

7. Arshad, M. U.; Gan, Y.; Wei, C.; Li, J.; Wu, C.; Naraghi, M., Fracture Toughness Characteristics of Stacked Bilayer Graphene via Far-Field Displacement Measurements. *Small n/a* (n/a), 2302499.

8. Arshad, S. N.; Naraghi, M.; Chasiotis, I., Strong carbon nanofibers from electrospun polyacrylonitrile. *Carbon* **2011,** *49* (5), 1710-1719.

9. Rammerstorfer, F. G., Buckling of elastic structures under tensile loads. *Acta Mechanica* **2017,** *229* (2), 881-900.

10. Arshad, M. U.; Gan, Y.; Wei, C.; Li, J.; Wu, C.; Naraghi, M., Fracture Toughness Characteristics of Stacked Bilayer Graphene via Far-Field Displacement Measurements. *Small* **2023,** *19* (39), e2302499.

11. Wei, X. D.; Naraghi, M.; Espinosa, H. D., Optimal Length Scales Emerging from Shear Load Transfer in Natural Materials: Application to Carbon-Based Nanocomposite Design. *Acs Nano* **2012,** *6* (3), 2333-2344.

12. Sakhavand, N.; Shahsavari, R., Universal composition–structure–property maps for natural and biomimetic platelet–matrix composites and stacked heterostructures. *Nature Communications* **2015,** *6* (1), 6523.
